# Convergence in the size and frequency of macroevolutionary peak-shifts on the adaptive landscape in vertebrates

**DOI:** 10.1038/s41467-026-78289-z

**Published:** 2026-10-27

**Authors:** Mark Pagel, Ciara O’Donovan, Andrew Meade

**Affiliations:** School of Biological Sciences, University of Reading, Reading, UK

**Keywords:** Phylogenetics, Evolutionary genetics

## Abstract

Empirical and theoretical support is emerging for a view of macroevolution characterised by species’ phenotypes tracking continually-moving adaptive peaks, interspersed with rare large shifts in the position of those peaks, likely corresponding to changes in species’ adaptive zones. But we lack understanding of the factors that control these movements. We show in a sample of over 21,000 species from the five Vertebrate Classes (*Mammals, Birds, Reptiles, Fish and Amphibians*) repeated convergence in the magnitude and frequencies of rare shifts in body-size peaks among species inhabiting the same dominant ecology. Peak-shifts (towards becoming larger or smaller) have been largest and most frequent among aquatic species, followed by terrestrial and then smallest and least frequent among volant species, independently of phylogenetic history. All peak-shifts are small in macroevolutionary time indicating that species in the terrestrial and aerial environments could have changed more had their environments favoured it. Within-population trait-variances from independent sources are largest in aquatic, followed by terrestrial then aerial species, consistent with evolution responding to differing macroevolutionary landscapes. Our results illustrate how ecologically varying adaptive landscapes have produced convergent outcomes of vertebrate macroevolution, giving rise to predictable evolutionary patterns even among species differing in fundamental aspects of development and thermoregulation.

## Abstract (web summary)

Biological evolution is often thought of as unrepeatable, dependent upon myriad historical contingencies. Contrary to this view, this study shows detailed similarities in the ways that vertebrate species adapt to aquatic, terrestrial and aerial environments despite being separated by millions of years of evolution.

## Introduction

Evolutionary biologists use the term macroevolution to refer to the processes that lead to differences among species and higher taxonomic groups in contrast to microevolution that refers to evolution that occurs within a species^1–3^. One of this field’s most enduring observations is that morphological change over evolutionary timescales—as witnessed in the fossil record and phylogenetic datasets—is often characterised by long periods of stasis during which little or no change occurs, but interspersed with shorter bouts of abrupt, episodic or punctuational change^1,3–13^. Empirical studies in large datasets^13,14^ and theoretical treatments^13–16^ of these phenomena point to macroevolution typically being bounded on short timescales—on the order of 1 million years or less—followed by divergence among lineages on longer timescales. Models attribute the bounded phase to species undergoing stabilising selection to perpetually moving but local adaptive optima. Over longer timescales, divergence is driven by a succession of rare but substantial shifts in adaptive optima, possibly corresponding to species entering new adaptive zones as Simpson envisaged^3^ in the 1940s.

These observations and models focus attention on the magnitudes and frequencies of the episodes of abrupt change as measures for understanding the dynamic landscapes to which species adapt over macroevolutionary time, and how species diverge phenotypically. A central challenge for the study of macroevolution, then, is to provide explanations, rooted in microevolutionary theory, for historical differences among lineages in the magnitude and frequencies of these abrupt shifts to new adaptive zones. Or as Pennel and Jiang^17^ have asked “Can we make predictions about the shape of the macroevolutionary adaptive landscape as well as how populations might move across it?”

Species’ macroevolutionary divergence is often observed to evolve along genetic lines of least resistance, roughly corresponding to the axis of maximum genetic variance between two or more traits^18,19^, or to be greater in directions with more mutational variation^20^. This observation has been interpreted as suggesting that species’ abilities to respond to adaptive demands over macroevolutionary time are constrained by the availability and orientation of the relevant genetic variance-covariance structures. But as the authors of these and other studies acknowledge, it is difficult to maintain this view in light of the relatively small amounts of phenotypic divergence that are typically observed over macroevolutionary time, small enough to be accounted for by neutral drift using the input from mutation^20–22^, a finding we have previously shown also to be true in the Mammals^12^. In addition, there is a recognition that the genetic variance-covariance structures that give the appearance of trait macroevolution following lines of least resistance may in fact arise as a result of natural selection for phenotypic divergence rather than acting as its cause^18,23–26^, and theoretical and simulation studies^23,24^ predict this outcome. In this context, Arnold^16^ proposes that species diverge along “selective [as opposed to genetic] lines of least resistance” or where stabilising selection is weakest. This state of affairs has led Schluter^26^, discussing the “surprisingly successful” enterprise of predicting macroevolution from microevolution, to lament that “the true explanation—whether caused by genetic constraints or by the shape of the adaptive landscape—still eludes us”.

These contrasting perspectives also apply to macroevolutionary divergence in single traits. In particular, do differences among species in the magnitudes or frequencies of macroevolutionary shifts to new adaptive optima arise from differences in the availability of variance for selection to act on or does the shape of adaptive landscapes sculpt both this variance and determine the macroevolutionary profiles? There are more than 60,000 vertebrate species (mammals, birds, reptiles, fish and amphibians), ranging in size from a few grams to over 125,000 kg (a 50 million-fold or more range), whose dominant ecologies are to live in the air, on land or in the water. Bird, reptile and amphibian species have occupied at least two of these ecological settings more than once, and mammals have occupied all three. This, then, provides a phylogenetic opportunity analogous to a ‘common-garden experiment’^8^ to ask whether species inhabiting these three ecologies produce reliably different profiles in the magnitude and frequency of rare large macroevolutionary jumps in phenotypic traits, here body size, and do so independently of evolutionary history, that is, of the ancestral habitat of their Class. To the extent they do, features of the environments species move to—not from—are implicated in the tempo and magnitude of long-term macroevolutionary peak shifts among vertebrate species, and can provide insights into how environmental variables may differentially structure the adaptive landscape.

We study these two characteristics—the magnitude and the frequency of bouts of episodic change—using the Fabric model, a Bayesian, phylogenetic-comparative statistical method^12,27^ (Methods, Statistical modelling of trait evolution) that characterises the evolution of a phenotypic trait over long periods of time as a function of a Brownian diffusion process (an unbiased random walk) augmented by the abrupt appearance over time of new optima. These abrupt changes, denoted in the model by the parameter β, identify branches in the tree in which a directional shift in the phenotypic trait is inferred to have occurred. We refer to these macroevolutionary changes as peak shifts in the adaptive landscape, although we acknowledge this is an inference from patterns in the comparative data. The β can be interpreted as lineages responding to new species-specific optima (the new peak), which the model assumes species adapt to instantaneously in macroevolutionary time. Macroevolutionary changes tend to be small^12,21,22^ and we agree with Houle et al.^20^ who say that “mutational variation can easily fuel orders of magnitude more evolutionary change than is observed on geologic timescales”.

Independently of peak shifts, the Fabric model identifies changes throughout the tree in the size of the incremental steps of the Brownian diffusion. The Brownian variance, denoted by ${\sigma }^{2}$, can either be increased or decreased by a scalar quantity, υ, that can vary independently of changes to optima, and in previous work we have found these two processes to be distinct^12^. In this regard, the Fabric model differs from models of the Ornstein-Uhlenbeck process^28,29^ that presume a functional linkage between the variance of evolutionary change and how far a trait is from its evolutionary optimum: variance is smallest when traits are furthest from their optima, rising to its maximum only as the trait approaches the optimum. Similarly, as we have previously described^12^ models based on the Levy process^9^ assume that jumps in the position of the optimum have their own generating process and variance. By allowing these two processes to operate independently the Fabric model can detect a variety of patterns of phenotypic macroevolution^12^ in response to patterns in the data.

Our data comprise logarithmically-transformed (base 10) body sizes for 21,374 species drawn from the five Vertebrate Classes and arrayed on Class-level phylogenies obtained from the Time Tree: Timescale of Life^30,31^ (Methods, Phylogenetic trees and body size data). Species are categorised by their dominant ecology (aquatic, terrestrial or volant; Methods, Supplementary Data), and, for purposes of statistical control, by their thermoregulatory mode (endothermic or ectothermic; Methods, Supplementary Data).

We additionally analysed data from independent sources^32–35^ on within-population variances for a variety of Vertebrate species and phenotypic traits. We use these data to test the idea that any differences among the dominant ecologies in the patterns of macroevolutionary change will correspond to differences in within-population variances of species in these habitats, where we treat the latter as an indicator of the strength of stabilising selection.

## Results

All datasets favour a model incorporating Brownian motion augmented by rare large peak-shifts over a simple Brownian motion model and over a white-noise^13,14,16^ version of the peak-shifts model. In the latter model, local optima are treated as stable, and Brownian variance does not accumulate with time (Methods: Statistical modelling of trait evolution, Supplementary Table1). The improvement of the Fabric model over its white-noise version is consistent with the view that species are tracking continually moving adaptive peaks.

**Fig. 1:**
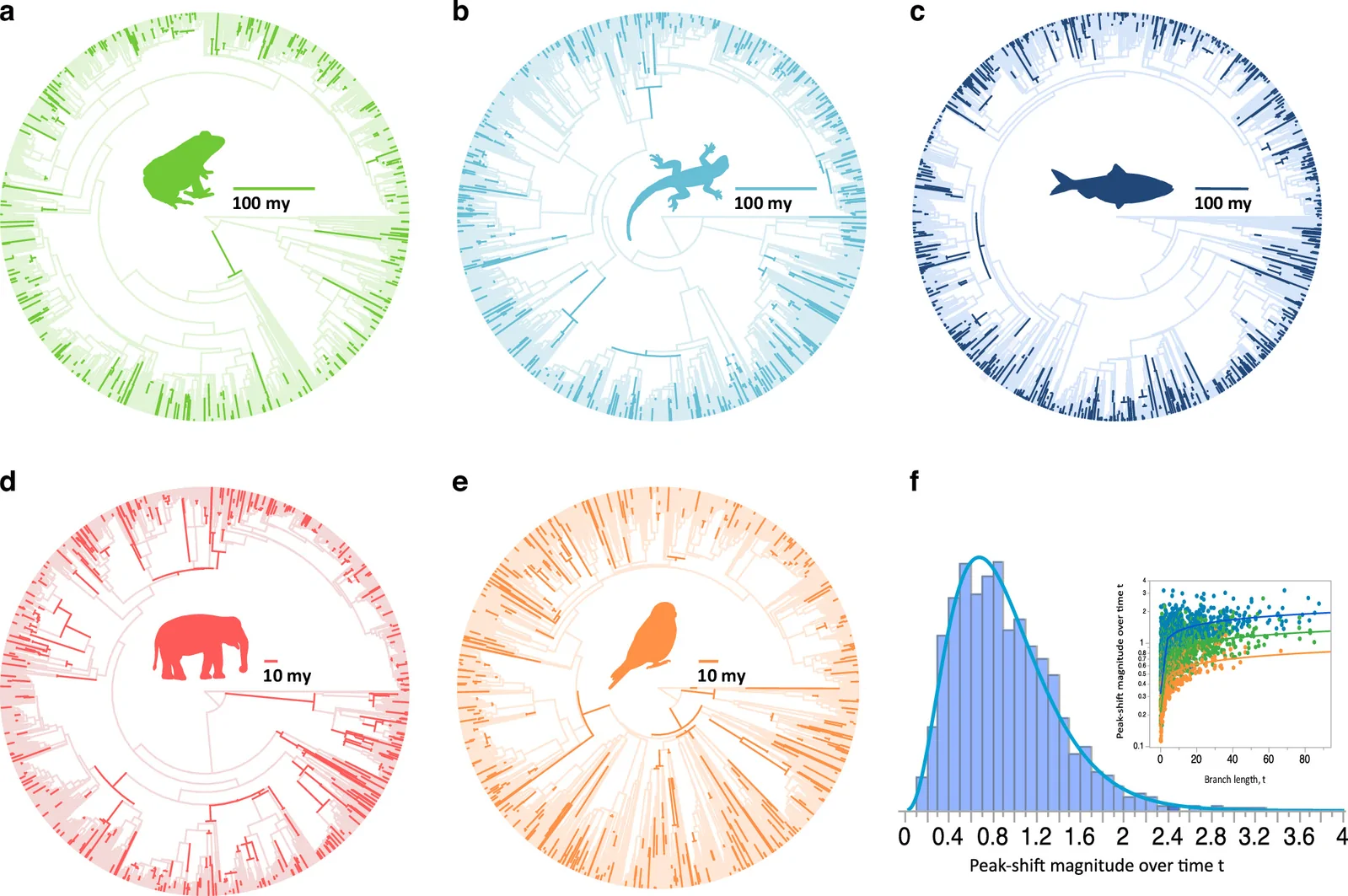
Occurrence of peak-shifts (β; n=2408) is widely distributed throughout the Vertebrates. Phylogenies of **a** Amphibians, **b** Reptiles (Lepidosaurs), **c** Fish (Actinopterygians), **d** Mammals and **e** Birds with branches in bold identifying branches in which a peak-shift occurred, **f** Histogram of the absolute peak-shifts (β) over time *t* with fitted Gamma curve; Inset Figure: Magnitude of peak-shifts over length of branch versus the length of the branch in which the peak-shift occurred. Blue dots correspond to peak-shifts in aquatic settings, green correspond to terrestrial ecologies and orange to aerial. Best-fitting log-log curves showing an asymptotic tendency (model fit R^2^ = 0.61, all ecological slopes differ at *p* < 0.0001, two-tailed Tukey’s HSD, *n* = 2408). All silhouettes are downloaded from www.phylopic.org and are classified as public domain under the CC0 1.0 Universal Public Domain Dedication license. Source data are provided as a Source Data file.

The peak-shift model identified 2408 instances of large shifts in size throughout the history of the Vertebrates (Methods, Posterior distributions), widely distributed among the five vertebrate Classes (Fig. 1a-e). Peak shifts occur in approximately 5.9% of branches, comparable to figures we^12^ and Hunt^7^ have found, although lower than an inference from Uyeda et al.^13^, indicating a pattern of change and stasis throughout vertebrate evolution. We do not observe a relationship between the absolute size of peak-shifts (a measure of how far the optimum has moved) and concomitant changes to the variance of evolution as measured by the $\upsilon \,$(*r* < 0.025, p > 0.21, 95% CI = −0.015, 0.065, df=2406), consistent with these being distinct processes. We do not analyse changes to the variance further, treating them as nuisance parameters relevant to model fitting. As previously observed in mammals^12^, the peak-shift model generally estimates ${\sigma }^{2}$ to be around 4-fold (Supplementary Table 1) smaller than in the Brownian model, meaning that up to 75% of the phenotypic divergence that Brownian models attribute to random walks, the Fabric model identifies as attributable to peak-shift processes. This illustrates the importance of peak shift processes in understanding macroevolutionary divergence.

Positive peak-shifts (increases in size) were slightly more common than negative shifts (1277 [53%] vs. 1131 [47%], ±24.54, binomial *p* = <0.0028, *n* = 2408; all significance tests we report are two-tailed). Unlike the simple Brownian model^36^, the Brownian+peak-shifts model can estimate ancestral values that deviate from the midpoint of the data. We do not analyse the positive and negative changes further, but convert effects to absolute values. Nevertheless, we control for the sign of the effect in all analyses, and none of our conclusions is changed by considering positive and negative changes separately.

We denote the magnitude of the peak-shifts by the parameter β, scaled to measure change per unit time (millions of years) along the phylogenetic branches to control for differences among lineages in branch lengths. The distribution of the 2408 shifts is right-skewed with a median of 0.129 (interquartile range (IQR) 0.066–0.300; geometric mean = 0.155; Fig. 1f). Positive changes (median = 0.115) are slightly smaller than negative changes (median = 0.142, *p* < 0.001).

The median β-change among the Vertebrates translates to 10^0.129^ = 1.35-fold change (IQR 1.17–1.98) in size per million years. An ancestral species weighing 1000 grams and experiencing a 1.35-fold size-increase along a branch of a million years would weigh 1350 grams, or an increase of approximately 3.5 × 10^−^^4^ grams per annum or 1.35 × 10^−^^3^ grams per generation conservatively assuming a generation time of four years.

### Magnitude of peak-shifts is only weakly related to time

The magnitude of the 2408 peak-shifts over the length of the branch in which they occurred rises rapidly with increasing branch-length and then approximately asymptotes, and does so separately within ecological categories (Fig. 1f inset). Some of the largest-magnitude changes occur in some of the shortest branches (Fig. 1f, inset), consistent with previous work suggesting that peak shifts typically occur in branches of at least 1 million years (far left of x-axis)^13^. Assuming peak-shifts are Poisson distributed and using the estimate of peak-shift frequency from this or from previous work^13^, the expected number of peak-shifts per branch is ~0.5, with a median = 0, suggesting that most of the peak-shifts represent a single event (we cannot rule out the possibility that change accumulates gradually and at its own pace in every branch but this explanation struggles with the asymptotic behaviour). This makes it plausible that peak-shifts coincide with speciation followed by long periods of relative stasis, a relationship we have previously observed in genes^10^, languages^37^ and morphology^12,38^ and consistent with punctuational change^11^.

### Peak-shift magnitudes differ among ecological categories

Our principal interest is in differences in the magnitude and frequency of occurrence of peak shifts as a function of a species’ dominant ecology. We tested for differences in the magnitude of peak-shifts (∣β∣) among the dominant ecologies (Aquatic, Terrestrial, Volant; Methods, Ecological categories) using a general linear model (Methods, Statistical analyses), controlling for differences among the Classes (Mammals, Reptiles, Birds, Amphibians and Fish), mode of thermoregulation (ectothermic/endothermic), the length of the branch in the phylogeny in which the change occurred, the historical timing of the episode measured as millions of years in the past, the sign of the change, and its interaction with ecology. We then estimated marginal mean effects for ecology, holding constant the effects of background variables.

**Fig. 2:**
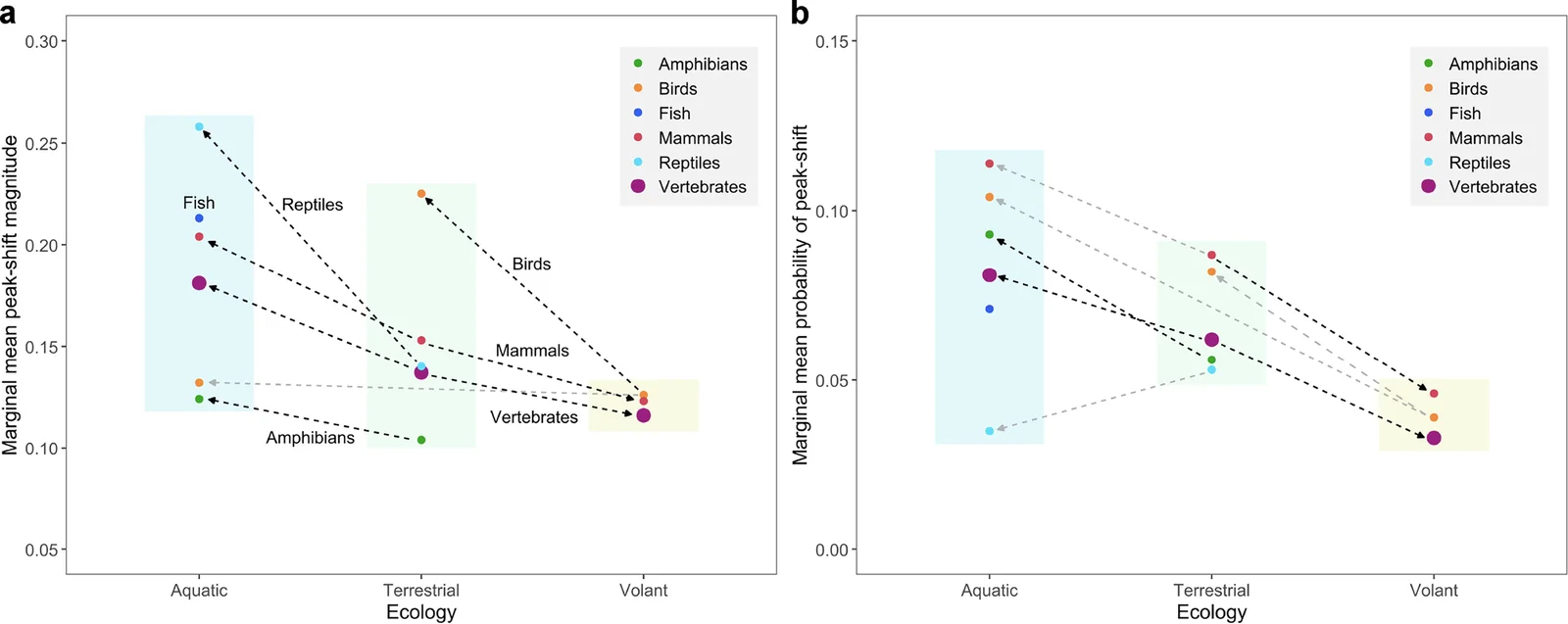
Convergence in the Peak-shift magnitudes and frequencies by Ecology. **a** Values for Vertebrates derived from full dataset of *n* = 2408 peak shifts. Values for Classes are derived from models fitted separately within each Class. Marginal mean peak-shift magnitudes are in units of change per million years on logarithmically transformed data. Arrows illustrate the presumed direction from ancestral to derived states in some Classes. All differences denoted by darker arrows are significant at *p* < 0.03 or less, two-tailed Tukey’s HSD tests (Supplementary Table 3). The large terrestrial mean for birds *n* = 3 transitions is influenced by two unusually large effects in the ratites (see text); **b** Marginal mean frequencies record the probability of a peak-shift occurring in a branch. Arrows point from an assumed ancestral state to the derived state. Darker arrows indicate a significant difference at *p* < 0.03 or less (Supplementary Table 4), pale arrows are not significant; marginal probabilities for aquatic and terrestrial birds can be estimated because the probability calculation counts all branches. Source data are provided as a Source Data file.

Across the Vertebrates the model yields *R*^*2*^ = 0.940 (*p* < 0.0001; Supplementary Table 2a). Peak-shifts are around 25% larger in aquatic environments than in terrestrial environments which in turn exceed by around 15% those in volant or aerial environments (Fig. 2a, Supplementary Table 3, *p* < 0.0001, all pairwise tests are two-tailed and use the Tukey HSD criterion that controls for multiple comparisons). If these peak shifts represent movements to new adaptive zones, then successive adaptive peaks are further apart in aquatic settings than in terrestrial environments and, in turn, further apart than successive peaks in aerial environments.

We find the same effects of ecology estimating the model’s parameters independently within each of the Classes (Fig. 2a, Supplementary Tables 2b–f, Supplementary Table 3). Aquatic peak-shifts are larger than those in terrestrial environments, and aerial peak shifts are smaller, including several cases of detailed convergence among Classes: peak-shifts in aquatic mammals (a derived ecology for mammals) are of very similar magnitude to those of the fish (ancestral ecology), and they exceed those in mammals’ own terrestrial habitat (mammals’ ancestral habitat, *p* < 0.003); in turn, volant mammals have smaller peak-shifts than terrestrial mammals (*p* < 0.0005) and are nearly identical to those of the birds. Peak-shifts are significantly larger in aquatic reptiles and aquatic amphibians than those of their Class living in the ancestral terrestrial environments (*p* < 0.001, *p* < 0.025 respectively).

Like the mammals, birds have occupied all three environments. The small number of avian aquatic (*n* = 3) and terrestrial (*n* = 3) peak-shifts yield a significant difference in birds only between terrestrial and volant forms. The large terrestrial mean peak-shift arises from the exceptionally large increase in size along the branch leading to the ratites from their volant ancestors, followed by a large reduction in size within this lineage along the branch leading to the kiwis (*Apteryx sp*). These small ratites are thought to have descended from the elephant birds of Madagascar (*Aepyornithidae*)^39^. The birds’ relatively small aquatic peak shifts, although larger than the volant peak shifts, are closer to typical terrestrial peak shift magnitudes. This may be because they occurred in the penguins whose life histories are divided between water and land.

We can separate the peak shifts in Fig. 2 into those that occur among species or lineages residing in their ancestral habitats (‘ancestral ecology’ peak shifts) and peak shifts that occur among species or lineages residing in a habitat that differs from the ancestral habitat of their Class (‘derived ecology’ peak shifts). There are 95 derived ecology peak shifts encompassing 304 species. Their magnitudes converge upon those of the ancestral ecology’ cases (Table 1), as well as of the Vertebrates as a whole. The derived-state lineages in Table 1 include the fruit and insectivorous bats among volant mammals; sea lions, seals, porpoises, dolphins, toothed and baleen whales among aquatic mammals; the emperor and little penguins among aquatic birds, the terrestrial flightless birds including cassowaries, ostriches, rhea, kiwis and rails; sea snakes among aquatic reptiles and salamanders, frogs, toads and newts among the aquatic amphibians (for the full list see Supplementary Data Table 1).

**Table 1:** Occurrence and marginal mean peak shift magnitudes of ninety-five instances of species or lineages (comprising n = 480 species) that inhabit a derived ecology for their Class and exhibit significant peak shifts, compared to species that have remained in their ancestral habitats, by Class and Ecology

	Aquatic	Terrestrial	Volant
	*Frequencies of derived cases by Class*		
Amphibians	15		
Birds	3	3	
Mammals	13		56
Reptiles	5		
Total	36	3	56
	*Marginal mean peak shifts by Ecology*		
Marginal mean peak shift and 95% CI for species and lineages in a derived ecology, *n* = 95	0.189 0.166-0.215 *n* = 36	0.174* 0.112-0.272 *n* = 3	0.117 0.096-0.142 *n* = 56
Marginal peak shift and 95% CI for species and lineages in their ancestral ecology, *n* = 2313	0.209 0.181-0.237 *n* = 806	0.139 ± 0.0097 0.120-0.158 *n* = 1076	0.099 ± 0.017 0.065-0.132 *n* = 431

^*^ Excluding a large peak shift associated with the cassowaries, rhea and ostriches which achieved exceptionally large sizes this marginal mean falls to 0.150 (95% CI = 0.09–0.244).

Notes: The numbers in the upper part of the Table record the number of independent lineages or species that occupy the ecology shown and which is derived for their Class. The marginal means are calculated from regressions as in Fig. 2. In the derived analyses thermoregulation is collinear with Class and so is excluded. Volant marginal peak shifts in the derived ecology dataset differ from terrestrial and from aquatic (pooled variance Tukey’s HSD *t*test, two-tailed); neither differs from terrestrial (*n* = 3, all flightless birds, including cassowary, rhea and ostriches). Class and ecology are collinear in the ancestral ecology dataset so the marginal means cannot be separated from Class: Aquatic = Fish, Terrestrial=Mammals, Reptiles and Amphibians, Volant=Birds. For species in their ancestral ecology Aquatic peak shifts > Terrestrial > Volant.

### The frequency of peak shifts differs among ecological categories

We fitted logistic regressions to binary data indicating the presence or absence of a change in a branch to test whether the probability that a branch would experience a peak shift varied with ecology. As before, our analyses control for Class, thermoregulation, branch length, time in the past and sign of the effect. We then calculate the marginal probability of a change in a branch. Across the Vertebrates, the marginal frequency of peak-shifts was highest in aquatic environments (Aq=8.1% of branches), followed by terrestrial (Te=6.2%, *p* = 0.025 vs Aq) and lowest in aerial environments (Vo=3.3%, *p* < 0.0001 vs Aq and Te), mirroring the effect of ecology on the peak-shift magnitudes (Supplementary Table 4, Fig. 2b).

Fitting the logistic model independently within Classes (Fig. 2b), the direction of effects is the same as for the Vertebrates as a whole within birds, mammals and amphibians (note: because the frequency analyses count all branches in the phylogeny, we can calculate frequencies for the birds in all three habitats in spite of few β changes). Aquatic peak-shifts occur significantly more frequently than terrestrial in amphibians (p < 0.03) and aerial peak-shifts occur significantly less often than both aquatic and terrestrial shifts in mammals (p < 0.001). Although smaller, the reptile aquatic peak-shift frequency does not differ from its terrestrial frequency; the small number of peak-shifts in this category (*n* = 5) makes this parameter difficult to estimate. It might also be that the frequency of change was higher in the past for the aquatic reptiles, as this group contained many large and now extinct lineages^40^.

As with the peak-shift magnitudes, we can study the probability of a peak shift occurring in the derived versus ancestral ecology branches. There are 1880 derived-ecology phylogenetic branches across the five Classes. With the possible exception of terrestrial derived forms, we observe convergence in the probabilities of a peak shift occurring along these derived ecology branches compared to the remaining branches for which the species or lineage occupies a habitat that is ancestral (ancestral ecology, Table 2). The relatively high probability in the derived terrestrial lineages (flightless birds) may reflect the numerous independent opportunities for flightlessness to evolve in this group with subsequent increases in size^41^. More generally, and across all three ecologies, these derived-state lineages may underestimate the latent evolutionary potential (probability) that resides in vertebrate species to change their dominant habitats. What might be termed partial or ‘probing’ transitions may be occurring among the so-called flying and walking fish and the gliding mammal, reptile and amphibian species.

**Table 2:** Occurrences and marginal probabilities of a peak shift per branch for n=1880 instances of species or lineages that inhabit a derived ecology for their Class, compared to species that have remained in their ancestral habitats, by Class and Ecology

	Aquatic	Terrestrial	Volant
	*Frequencies of derived cases by Class*		
Amphibians	165		
Birds	31	25	
Mammals	263		1279
Reptiles	117		
Total	**576**	25	1279
	*Marginal probabilities of change per branch by Ecology*		
Marginal probability of change in a branch, derived ecology, n = 1880	**0.095**, *n* = 576 Vo	**0.119**, *n* = 25 Vo	**0.046**, *n* = 1279 Vo
Marginal probability of change in a branch, ancestral ecology, n = 38869	**0.079**,*n* = 9690	**0.061**,*n* = 16922	**0.031**,*n* = 12257
	Te,Vo	Aq,Vo	Aq,Te

### Peak-shifts are small on a macroevolutionary time-scale

**Fig. 3:**
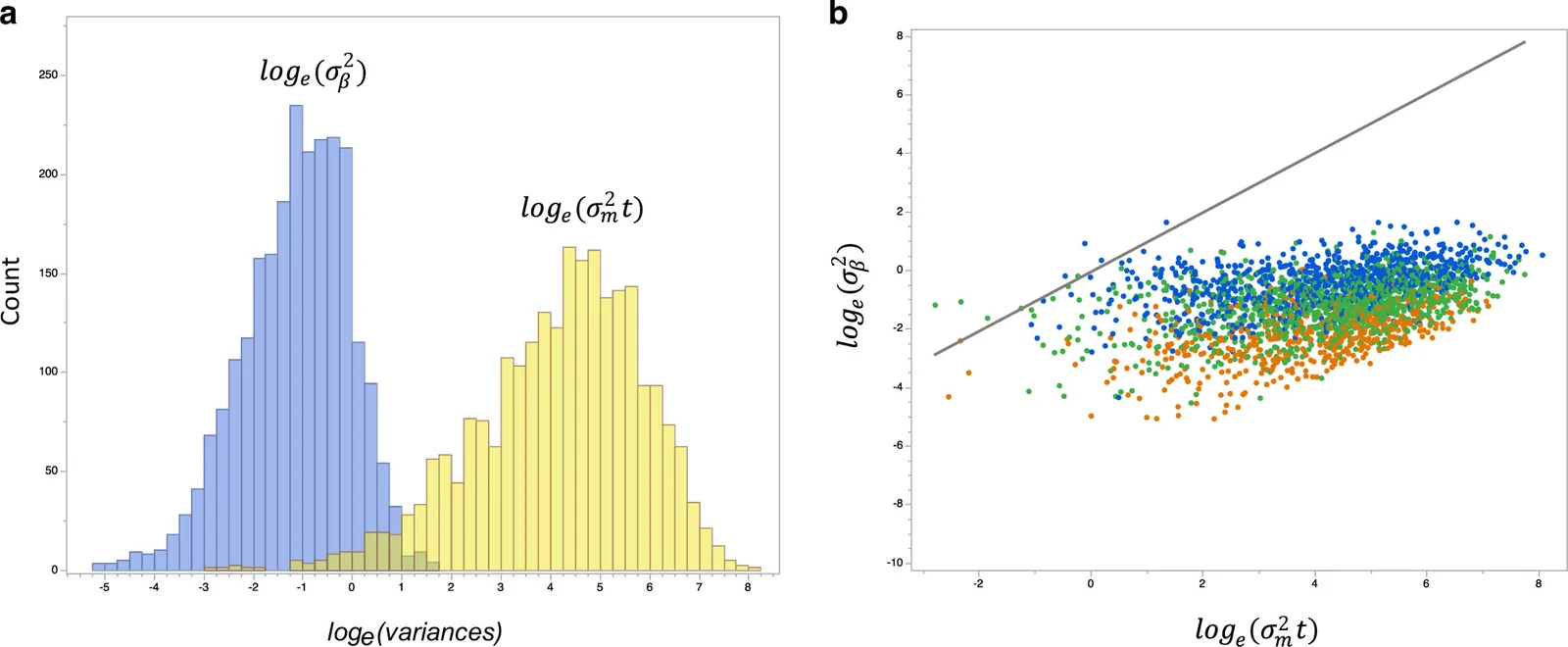
Measured variance of peak-shifts versus mutational variances. **a** The measured variances of change along the branches attributable to peak-shifts (*n* = 2408, see text; blue histogram) are smaller than an estimate of the mutational variance that would be available along the same branches (yellow histogram; Methods, Estimation of variance arising from mutation). Sufficient mutational variance exists for drift to produce the observed large changes; **b** Same data plotted as variances of peak-shifts on *y*-axis versus expected mutational variance on *x*-axis. Line is 1:1 relationship showing that observed variances of change are smaller than are available by neutral drift, separately for the three different dominant ecologies (blue=aquatic, green=terrestrial, orange = aerial). Source data are provided as a Source Data file.

The peak-shifts include movements towards getting smaller or larger, and even the largest, such as observed in the aquatic species, are small in macroevolutionary time as discussed previously in connection with the distribution of β effects. Following Lynch^22^, we asked whether the peak shifts are small given the amount of change that could be expected from the accumulation of new variance attributable to mutation. We generated the expected variance of change in each branch attributable to mutation from ${\sigma }_{m}^{2}t$, based on surveys of mutational variances^42,43^ (${\sigma }_{m}^{2}$) and assuming neutral drift over the period of time (*t*) represented by the length of the branch in which the peak shift occurred (Methods, Estimation of variance arising from mutation). We compared these values to the observed variance of change as implied by the β (analogous to an ancestor-to-descendant between-groups variance) in the same branches and given by ${\sigma }_{\beta }^{2}$ (Methods, Estimation of variance arising from mutation; Fig. 3a,b).

Observed amounts of change are small compared to what could be achieved by drift acting on the mutational variances, and this is true in all three ecological settings. Houle et al.^20^ similarly concluded that *Drosophila* wing macroevolution has not been limited by the availability of mutational variance. The significance of this in interpreting the marginal mean effects by ecology is that species in terrestrial and aerial environments could have produced changes comparable in magnitude to those we observe in aquatic species had they been favoured^12,20,22,28,44^.

### Within-population variances differ among ecological categories

We obtained data on within-population variances from two independent compilations^32,33^ of phenotypic traits in animal and plant populations and used them to compare average variances in body size or size-related traits among the ecological categories restricting our samples to vertebrate species (Table 3, sources in Supplementary Table 5). Terrestrial species in Table 3 include mammals, reptiles and amphibians, but most aquatic species in these two compilations are fish and the volant species are birds so we augment these data with two additional compilations, one in megachiroptera^34^ (fruit bats) and one in cetacea^35^.

**Table 3:** Within-population geometric mean variances of phenotypic trait data in the Vertebrates categorised by habitat, drawn from independent meta-analyses and field datasets

Dataset	Habitat			
**Source 1**^32^, Geometric mean ±1 s.e.m.	**Aquatic** 0.015 0.013-0.018 *n =*14 (127)	***Terrestrial*** 0.007 0.006-0.008 *n* = 14 (104)	***Volant*** 0.003 0.0025-0.0034 *n* = 17 (186)	**No. species** **(populations)** *n* = 45 (417)
Size traits only, Geometric mean ±1 s.e.m.	0.011 0.008-0.014 *n* = 8 (19)	0.005 0.004-0.006 *n* = 11 (66)	0.0016 0.0014-0.0018 *n* = 14 (168)	*n* = 33 (253)
**Source 2**^33^, Geometric mean ±1 s.e.m.	0.012 0.011-0.013 *n* = 7 (231)	0.0076 0.0069-0.0083 *n* = 11 (262)	0.0023 0.0019-0.0026 *n* = 15 (194)	*n* = 33 (687)
Size traits only, Geometric mean ±1 s.e.m.	0.009 0.008-0.011 *n* = 6 (112)	0.0045 0.004-0.005 *n* = 9 (200)	0.0020 0.0018-0.0023 *n* = 13 (147)	*n* = 28 (459)
**Cetacea**^35^ **and Megachiroptera**^34^, Geometric mean size ±1 s.e.m	0.0097 0.0081-0.0012 *n* = 21 (21)		0.00075 0.00068-0.00083 *n* = 37 (37)	*n* = 58 (58)

Notes: Selection criteria. Data from sources 1 and 2 is restricted to vertebrate populations with coefficients of variation ≤ 0.3, and which had additive genetic variances >0 (Methods). Sample sizes refer to the number of species (no. populations in parentheses); See Methods for full selection criteria and Supplementary Table 5. Pairwise significance tests conducted on log-transformed data with species nested within ecological categories to control for multiple populations of the same species. Dataset 1: All ecological pairs averaged over all traits or restricted to morphology traits only are significantly different on Tukey HSD at p<0.001 or less two-tailed (save for Aq-Te difference restricted to morphology, *p*<0.05 two-tailed HSD test); Dataset 2: All ecological pairs averaged over all traits or restricted to morphology traits only are significantly different on Tukey HSD at p<0.0009 or less two-tailed. The Cetacea and Megachiroptera datasets differ at p<0.0001 (two-tailed). Data publicly available in refs. ^32, 33, 34, 35^.

Geometric mean phenotypic variances within populations (data log-transformed to remove scaling effects and controlling for replicate populations of the same species) are largest in aquatic species, followed by terrestrial and smallest among volant species matching the rank-order of the macroevolutionary peak-shift magnitudes and frequencies (Table 3). Larger within-population variances within the fish may reflect indeterminate growth, but we find similarly large variances within the cetacea. The megachiroptera are similar to other volant species.

To the extent these within-population variances are representative of their environments, they provide a platform to link micro-evolutionary processes to the macroevolutionary differences in the magnitude and the frequency of peak-shifts. Assuming genetic variances are proportional to the phenotypic variances, their rank ordering by ecological category (Aq>Te>Vo) is consistent with weaker stabilising selection (wider fitness curves) and larger genetic variance in aquatic species compared to terrestrial and in terrestrial compared to volant species. Wider fitness curves mean that individuals can vary more within a species without significant loss of fitness. But this also means that for a population to move to a new adaptive zone, that zone must be farther away (a larger peak shift) for natural selection to overcome ongoing gene flow arising from drift and interbreeding with the source population. At the same time, weak stabilising selection enhances the probability or frequency of movement to new optima owing to increased stochastic variation of population means (Lande^21^, eq. 20). Or conversely, Lande^21^ (Lande’s Fig. 2) showed that even small increases in the strength of stabilising selection (yielding smaller trait variances) can lead to a large increase in the time taken for a population to drift across the threshold into the pull of another adaptive zone.

## Discussion

We find that the habitats of earth, air and water are consistently associated with different macroevolutionary profiles in the magnitudes and the frequencies of repeated and independent shifts in the positions of adaptive peaks. Successive adaptive peaks are further apart in aquatic environments than in terrestrial environments, which in turn are further apart than in aerial environments, and analyses show that species in these latter two habitats could have responded to larger peak shifts in macroevolutionary time had their environments favoured it. Species that move into a new habitat—such as the cetacea, seals and sea lions, aquatic reptiles and amphibians, flightless birds, or the chiroptera—acquire peak-shift profiles that are convergent on those of species who occupy those habitats ancestrally (fish, terrestrial mammals, reptiles and amphibians, and birds) in spite of millions of years of divergence in development, thermoregulation, life histories and the accumulated caprices of history. Our results extend in time and phylogenetic breadth observations of morphological and behavioural convergence documented among lizards from the genus *Anolis*^45^ to comparisons among vertebrate Classes.

One explanation for our findings sees the convergence in macroevolutionary patterns of change as having been sculpted by the differing demands of the aquatic, terrestrial and volant environments; the other suggests that pre-existing differences in the availability of quantitative-genetic variance among species determine the course of macroevolution. Features of our data lead us to favour the former explanation. The pre-existing genetic variance view requires, in the current setting, the assumption that when species move from their Class’s ancestral ecological setting to a new derived one, such as when terrestrial mammals took to the water, or to the air, it was only those that had particular genetic variances—large for those moving to aquatic habitats, small for volant—which did so. While possible, the data we report on within-population variances makes this seem unlikely. And, even if the ancestors that initially moved had variances conducive to doing so, the tens to hundreds of millions of years species have spent in their derived ecological settings is far more than is needed for their phenotypic variances and covariances to have evolved (via selection and mutation) to reflect the demands of the setting^44^.

The finding of larger peak shifts in aquatic environments and smaller in aerial ones is not simply a restatement of well-known observations on the average size of species in different habitats. Some aquatic species, such as cetacea, are unusually large, but peak shift sizes for the aquatic mammals are no larger than those of the fish, which are generally small in size, as are the marine reptiles and amphibians. Conversely, although the maximum size of flighted species is constrained by gravity and the demands of flight, many birds and even some megachiroptera are larger in size than small terrestrial mammals, reptiles and amphibians but have smaller peak shifts on average.

Rather, we suggest that the differing magnitudes of macroevolutionary peak shifts arise from the differing shapes of the aquatic, terrestrial and aerial adaptive landscapes. Large within-population variances, such as in aquatic environments, suggest weaker stabilising selection for size, meaning that individuals can vary widely in size within a species without large differences in fitness. Weak stabilising selection within populations then means that, where successive macroevolutionary peaks yield new species, these peaks must be further apart in trait space for populations to maintain their genetic identities in the event of interbreeding. In aerial environments, the strong stabilising selection on size means that successive adaptive peaks in the macroevolutionary landscape can be closer together. Flighted species could produce larger peak shifts, but on average, they do not need to.

The importance of understanding the origin of the peak shift magnitudes in explaining phenotypic divergence over macroevolutionary time looms large: peak-shifts accounted for upwards of 75% of the variation among species. To predict macroevolution and by implication phenotypic divergence over long periods of time, then, we need, as Schluter^26^, Arnold^15,16^ and others^21,46,47^ have emphasised, to understand how and why the shapes of adaptive landscapes differ among ecological settings and how or whether these interact with differences among species in thermoregulation, development and other factors. This includes the nature of pre-existing variances and covariances among species’ traits as they can affect the rate at which species adapt in the short term, and maybe longer.

The three environments of earth, air and water confront species with profoundly differing evolutionary opportunities and challenges^48,49^. Water’s density favours the familiar, relatively one-dimensional ‘fusiform’ body shapes of swimming species both small and large (e.g., fish, Cetacea, penguins, aquatic snakes), to reduce both drag and the energy needed to move through a dense medium^48^. Water’s neutral buoyancy additionally may mean that individuals within a species can vary in size without much variation in fitness because changes to size need not be strictly accompanied by changes to other structural features of the body. The reverse will be true in terrestrial and especially aerial environments, where the requirements for flight may require precisely correlated changes to size in multiple dimensions. A terrestrial compared to aquatic existence requires more powerful circulatory systems and higher arterial blood pressure to counter the gravitational force^50^, and aerial environments require intense and often sustained aerobic and energetic outputs^51^. These features of terrestrial and aerial environments plausibly translate into generally stronger stabilising selection, which in turn allow macroevolutionary adaptive peaks to be closer.

The differing physical characteristics of the three environments may also contribute to differences in the dimensionality of these environments. Terrestrial environments may, in general, have greater fractal complexity than aquatic environments^52,53^. A fractally more complex environment might result in smaller changes to size becoming more distinctive ecologically and in their effects on fitness, because, in contrast to the fusiform shape, other morphological dimensions, such as size and shape of limbs, play stronger independent adaptive roles. Possibly this explains the relatively small magnitudes of peak shifts in the aquatic amphibians who, in some regards, behave like their terrestrial counterparts, but do so underwater rather than on land.

Aerial environments might be the most complex and selection pressures on morphology the most optimised of the three: there is the constant challenge of staying airborne whilst maintaining manoeuvrability and flight speed. Whether this gave birds an advantage over their doomed terrestrial dinosaur counterparts is unknown, but it may suggest that a typical niche-space is more compact (stronger stabilising selection) in aerial than terrestrial environments and in terrestrial compared to aquatic environments.

The topologies of the aquatic, terrestrial and aerial adaptive landscapes could also help to explain differences among the three habitats in the frequency of peak-shifts. The fusiform body shape common to aquatic environments may mean that body size changes are funnelled into a smaller number of morphological dimensions (roughly changes to the length and breadth of the fusiform shape), increasing the frequency with which evolution acts on size per se. If there is greater fractal complexity in terrestrial and aerial environments, then there might be more ways than size for the species that occupy those habitats to adapt, and therefore again fewer changes to size itself. Fractal complexity might help to understand why birds have been able to occupy all three major habitats, producing adaptations from flight to flightlessness and swimming, song, colour, beak morphology and plumage, despite comparatively few and relatively small peak-shifts in size.

In the opening lines of their influential paper critiquing what they called an “adaptationist programme” in evolutionary biology Stephen Gould and Richard Lewontin^54^ stated that “…organisms must be analysed as integrated wholes, with Baupläne so constrained by phyletic heritage, pathways of development and general architecture that the constraints themselves become more interesting and more important in delimiting pathways of change than the selective force that may mediate change when it occurs.” It is true, history matters, but at least for vertebrates, it hasn’t discouraged species from the lure of new and potentially profitable environments, and when they reach those environments, their macroevolutionary profiles converge in how frequently they occupy new adaptive zones and how far they move when they do so, despite their divergent evolutionary histories. Differences in the demands of aquatic, terrestrial and aerial environments, combined with the number of possible combinations of mutations that natural selection can try out over 450 million years of evolution within untold numbers of populations evolving along numerous independent lineages, have revealed something of the shape of these environments and how species move across them.

## Methods

### Phylogenetic trees and body size data

We obtained phylogenetic trees from the TimeTree of Life^30,31^ using the ‘Build a Timetree’ function of the timetree.org website. We used the search terms ‘Mammalia’ (*n* = 4183), ‘Reptilia: Lepidosauria’ (*n* = 4702) and ‘Aves’ (*n* = 7163). The Actinopterygian (ray-finned) Fish (*n* = 6840) and Amphibian (*n* = 3506) phylogenies were cropped out of a phylogeny of ‘Chordata’ (*n* = 26808). We converted all trees to nexus format, soft polytomies were collapsed and transformed into hard polytomies and any zero length terminal branches and their terminal taxa were removed from the trees.

We collected the body size data for all five Vertebrate Classes from Cooney and Thomas^55^. Mammal data were supplemented with data from Ernest^56^ and Jones et al.^57^. Bird and Mammal body sizes are both measured in mass units. Reptile, Amphibian and Fish body sizes are all measured in length units as is common for animals with indeterminate growth. Fish lengths are measured as their maximum length. Within Amphibians, the variation in body plans between the Orders means that their lengths are measured using snout-vent length (SVL) for Anurans and total length for Gymnophiona and Caudata. Likewise, within Lepidosaurs, snakes are measured by their total length, whilst the remainder are measured by their SVL. We converted all measures into either grams for masses or millimetres for lengths and logarithmically (base 10) transformed all data before use in comparative models. See Supplementary Data for trees and body-size measures.

Theory predicts that length varies allometrically with mass according *to* ${mass}\propto {{length}}^{b}$, where *b* = *3* is predicted and supported empirically (Supplementary Table 6). We therefore multiplied all β parameters estimated in the statistical model by three (multiply because the data have been log-transformed), where they were derived from length measures, to put them on a common scale with those derived from mass measures (equivalent to transforming the raw data). This transformation allows for comparisons among classes, but our conclusions do not depend on the transformation, as we show how our results hold within as well as across the Classes of vertebrates.

We assigned species’ data to the phylogenies by exact name matches. Where taxa in the tree remained without data, we checked the name in the tree for synonyms using the IUCN Red List website^58^ and the Catalogue of Life website^59^ and then matched names in the tree to their synonym’s data (checking that each synonym was only used to make one match). Where there were cases of the same body size attributed to multiple species within a single genus, we removed all but one of the species from the dataset. Finally, we cropped the phylogenies to the same dimension of their corresponding dataset, resulting in final dataset dimensions of 3399 mammals, 6404 birds, 3917 reptiles, 2435 amphibians and 5219 fish altogether yielding the evolutionary history of 21,374 Vertebrates.

### Ecological categories

We classified all vertebrate species in our sample as aquatic, terrestrial or volant, based on the environment in which each species spends most of its time or where major life requirements are fulfilled (Supplementary Data). To infer the ecological category of internal branches of the phylogeny (to match to peak-shifts) we first established a ‘base ecology’ for each of the Vertebrate Classes which described the dominant ecology within each Class (Mammals = terrestrial, Birds = volant, Amphibians = terrestrial, Reptiles = terrestrial and Fish = aquatic).

Using published sources, we then identified species or groups of species within each Class whose dominant ecology differed from the Class’s base ecology (Supplementary Data). Where a monophyletic group of species all shared a common ecology which deviated from the base ecology of their Class, all branches descending from their most recent common ancestor were marked with their shared ecology. The branch preceding their most recent common ancestor was labelled with the base ecology but marked as a ‘transition branch’. If a contemporary species has altered its ecology relative to the base ecology, then we label the terminal branch with the new ecology. Our results are robust whether these single taxon transitions are included in our analyses or not.

### Statistical modelling of trait evolution

We used the Fabric model^12,27^ to identify historical instances of peak-shifts in our sample of Vertebrates (see Code Availability). Peak-shifts are denoted in the model by the parameter β and occur along the branches of a phylogenetic tree. The magnitudes of the β are allowed to vary among branches, and there is no predetermined number or placement of these events in a phylogeny. The model also simultaneously estimates the ancestral value of the trait at the root of the tree (denoted by the parameter α) and the instantaneous Brownian variance (here in units of variance per million years). A parameter υ corrects for evolutionary changes to the Brownian variance^12^ throughout the tree and is here treated as a nuisance parameter relevant to model fitting.

In addition to the Fabric (peak-shift) model we fit a simple Brownian-motion model and a white-noise version of the peak-shift model. In the Brownian and peak-shift models, the Brownian variance increases with time according to ${\sigma }^{2}t$ corresponding to species tracking constantly moving adaptive peaks, interspersed in the peak-shift model by rare large changes to the position of peaks. In the white-noise model, ${\sigma }^{2}$ does not increase with time and corresponds to the expectation of a system in which species are tightly adapted to stable local adaptive peaks^13,14,16^, only moving in the event of a peak-shift.

### Model comparisons

The models were estimated on each of the five body-size datasets (Mammals, Birds, Reptiles, Amphibians and Fish) in a Reversible-Jump Markov chain Monte Carlo (RJ-MCMC) framework, with their posterior distributions sampled from the stationary Markov chain^12^. We ran 12 replicate RJ-MCMC chains to stationarity for each model, followed by a long period of widely-spaced sampling to acquire posterior samples of 1000 iterations. We randomly chose six of the 12 chains for each model, and estimated their marginal likelihoods using a stepping-stone sampler^60^. We compared the mean (of the six selected replicates) marginal log-likelihoods of the models using BayesFactors (see Supplementary Table 1).

### Priors on model parameters

Prior distributions for the peak-shift parameters (β) used to study changes to body mass were taken from our earlier work^12^. For length measurements, we modified the prior distributions for mass by the allometric relationship ${mass}\propto {{length}}^{3}$. Priors on ancestral body sizes (α) were based on literature sources that provided evidence on the natural history of each Vertebrate Class (Supplementary Table 7)

### Posterior distributions

Based on previous work^12^, we selected for inclusion in our posterior samples of peak-shift parameters those that appeared in the same place in the tree more often than expected by chance and in all six of the replicate RJ-MCMC chains. Average intercorrelations of the estimated parameters among the six chains were ~0.98–0.99 and so we averaged the parameters across chains. This yielded *n *= 2448 estimated peak shifts, of which we excluded 20 for being of ambiguous sign (some posterior samples indicate a shift towards a larger size, others towards a smaller size) and a further 20 for appearing on branches categorised as either semi-aquatic or a transitional branch from one ecological state to another. This left n = 2408 size-shifts that fell on either an aquatic, terrestrial or volant branch of the phylogeny (see Ecological categories).

### Statistical analyses

To investigate peak-shift magnitudes among the ecological categories, we rescaled the βs associated with body length data (reptiles, amphibians, and fish) to be βs associated with body mass. To re-scale we multiplied the logarithmically-transformed body length β’s by three owing to the cubic relationship between body mass and length (Supplementary Table 6).

All statistical analyses were conducted in JMP Pro 19 (JMP^®^, Version *Pro 19*, SAS Institute Inc., Cary, NC, 1989–2021). To test whether peak-shift magnitudes are dependent on ecology we ran standard least squares regressions (βs are already phylogenetically controlled) on the βs:

$${\log }_{10}\left|\,\beta \right| \sim {Class}+{\log }_{10}{branch}+{\log }_{10}{time}+{ecology} \\+{thermoregulation}+{sign}\left(\beta \right)+{sign}\left(\beta \right)\times {ecology}$$

Here ${\log }_{10}\left|\beta \right|$ is a vector of 2408 mean values from the posterior sample of mass-β (mammals and birds) and mass transformed length-β (reptiles, amphibians and fish). We show in the Supplementary Information that the estimated variance-covariance matrix of the β’s is diagonal with homoscedastic variances, allowing us to treat them as independent and identically distributed variates.

We logarithmically transformed the βs, branch lengths, and times in the past to linearise their relationships. *Ecology* and *thermoregulation* (ectothermic/endothermic) are coded in the model as nominal variables; *Class* is a nominal factor coding for Mammals, Reptiles (avian and non-avian), Amphibians, and Fish. Coding the Reptiles this way allows us to include thermoregulation as a separate factor in the regressions, otherwise it is confounded by taxonomic Class. This has no effect on our conclusions: the results are algebraically identical if we code the Birds and non-avian Reptiles as separate Classes. With that coding the effect of thermoregulation is subsumed in the comparison between the Birds and Mammals versus the remaining three Classes. *Branch lengths* refer to the length (in millions of years) of the branch of the phylogenetic tree on which the β occurred; *time* is the time (in millions of years) before the present at the middle point of the branch in which the directional change occurred. The latter is included to control for any changes in the magnitude of βs through geological time.

Owing to the uneven distribution of ecological and thermoregulatory traits across the Classes, we did not include interactions with the Group factor in the Vertebrates model but instead calculated the same regression separately within the taxonomic groups. Including interactions between branch length and time, and between the Class factor and each of these two variables, increased the overall *R*^*2*^ from 0.939 in the model without interactions (main text) to 0.940. The interactions have no effect on our conclusions.

We tested for differences in peak-shift magnitudes among the marginal means of the ecological categories using Tukey Honestly Significant Differences tests (two-tailed), which controls for Type I errors and simplifies to a Student’s t-test when only two categories exist. Marginal means sum over the equally weighted effects of categorical variables (such as Class), thereby removing them from the estimator, and hold constant background continuous variables. None of our conclusions from these analyses is changed if the 40 excluded branches (see Posterior distributions, above) are included in analyses of peak-shift magnitudes.

Tests for the frequency of occurrence of peak-shifts used the same framework as above, substituting logistic for linear regression. We include the 40 excluded branches in the peak-shift frequency analyses because they do represent events even if of less certain identity.

### Estimation of variance arising from mutation

Following Lynch^22^ we estimate the phenotypic variation arising over time from mutation as the product of the mutational variance, ${\sigma }_{m}^{2}$, and *t* where *t* is the length of time measured in millions of years. A common observation is that ${\sigma }_{m}^{2}$ as measured in mutation accumulation studies is on the order of 10^−4^ to 10^−2^ of the environmental variance ${\sigma }_{{env}}^{2}$ in a phenotypic trait^22,42^. We calculate ${\sigma }_{{env}}^{2}=({1-h}^{2}){\sigma }_{T}^{2}$, where ${\sigma }_{T}^{2}$ is the within-population variance of the logarithmically-transformed phenotypic trait, and ${h}^{2}$ is the narrow sense heritability. Here we use ${h}^{2}=0.33$ on the basis of recent meta-analysis that reported estimates of heritability for ~2600 observations on 170 species^32^; we set ${\sigma }_{T}^{2}=0.004$, which is the mean of the variances of the logarithmically transformed morphological traits in Table 1 (log-transformed to remove scaling effects).

Then, to estimate ${\sigma }_{m}^{2}t$ we drew a random $U \sim ({\mathrm{0.0001,0.05}})$ to capture the range 10^−4^ to 10^−2^, multiplied this value by $({1-h}^{2}){\sigma }_{T}^{2}$, and then by *t*. Finally, we scale this value by a conservative estimate of one generation per four years (250,000 per million). This approach to estimating the mutational variance will overestimate positive mutations by including both deleterious and beneficial mutations but by how much is debated^61,62^, and here our interest is to get a sense of how much variation assuming that most mutants are of small effect (i.e., neutral or nearly-neutral). Finally, we compare these estimates of variance arising from mutation to the observed variance of change owing to peak-shifts given by ${\sigma }_{\beta }^{2}=\frac{{(\beta t)}^{2}}{2}$ as we have shown elsewhere^12^.

### Within-population variances

We used data from studies reported in Hansen and Pèlabon^32^ and Kingsolver and Diamond^33^ excluding plants and invertebrate species, plus data on Megachiropterans^34^ and Cetaceans^35^ (see Supplementary Table 5). We excluded populations with CV > 0.3, where CV is the coefficient of variation $\sigma /\mu$ in the raw data, and additive genetic variances equal to zero. Population variances were converted to variance on a log-scale according to ${\sigma }^{2}$(ln(*x*)) ≅ CV^2^ for CV≤0.3. In the Cetacean and Megachiropteran datasets the within-population variance increased with the number of individuals sampled per species. We removed species with small sample sizes (<10 individuals sampled for megachiroptera, <30 for cetacea) until the positive association vanished and then calculated variances on the remaining populations.

### Reporting summary

Further information on research design is available in the Nature Portfolio Reporting Summary linked to this article.

## Supplementary information

Supplementary Information

Transparent Peer Review file

Reporting Summary

Description of Additional Supplementary Files

Supplementary Data 1

Supplementary Data 2

## Source data

Source Data

## Data Availability

Data are provided as Supplementary data files 1 and 2. Source data are provided as a Source Data file. Other data (Table 3) are derived from published publicly available datasets (see links in text and Supplementary Information). Source data are provided with this paper.

## Code availability

Analyses were run using BayesTraits version 4.1.1 which is freely available from https://www.evolution.reading.ac.uk/BayesTraitsV4.1.1/BayesTraitsV4.1.1.html
