# Supplementary material for: Convergence in the size and frequency of macroevolutionary peak-shifts on the adaptive landscape in vertebrates
Source: Nat Commun. 2026 Oct 27;17:11208. doi: 10.1038/s41467-026-78289-z (PMC13720082; doi:10.1038/s41467-026-78289-z)
Supplement: Supplementary file 1 [file 41467_2026_78289_MOESM1_ESM.pdf]

SUPPLEMENTARY INFORMATION

Convergence in the size and frequency of macroevolutionary peak-shifts on the adaptive landscape in vertebrates

Mark Pagel*, Ciara O'Donovan and Andrew Meade

Supplementary Table 1 Model Comparisons

Supplementary Tables 2a-f Results of General linear modelling

Supplementary Table 3 Marginal mean effects by Class and Ecology

Supplementary Table 4 Marginal probabilities of change by Class and Ecology

Within-population trait variances and Supplementary Table 5

Scaling of length measurements and Supplementary Table 6

Priors on Model parameters and Supplementary Table 7

The variance-covariance matrix of the peak-shift parameters' (β) and Supplementary Table 8

Supplementary References

Model comparisons

We estimated marginal likelihoods using a stepping-stone sampler (see Methods, main text) and found the average of twelve-marginal log-likelihoods obtained from independent Markov chains run to stationarity and then sampled at widely-spaced intervals. All datasets prefer the Brownian+Peak-shift model.

SI Table 1 Marginal likelihoods of Brownian, Peak-shift and white-noise Peak-shift models.

	Model					
	Brownian		Peak-shift+Brownian		White-noise peak-shift	
Class	Marginal likelihood	Brownian variance	Marginal likelihood	Brownian variance	Marginal likelihood	Brownian variance
Amphibians	610±0.03	0.0126	**1284±38**	0.0027	1069±153	1.8×10^{-4}
Birds	951±0.01	0.0046	**2430±142**	0.0015	2065±67	1.0×10^{-4}
Fish	-1722±0.01	0.0513	**1045±87**	0.0126	395±80	4.5×10^{-4}
Mammals	-1522±0.01	0.0123	**-261±32**	0.0027	-499±187	1.2×10^{-4}
Reptiles	108±0.01	0.018	**2138±39**	0.0027	1500±221	2.0×10^{-4}

Supplementary Tables 2a-f : Regressions

Note: all p-values reported in Tables 2a-f are two-tailed.

2a. Vertebrates. R^2 = 0.940

**Source**	**No. parameters**	**DF**	**Sum of Squares**	**F Ratio**	**Prob > F**
Class	3	3	2.3352	8.8482	<.0001*
Thermoregulation	1	1	5.4154	61.5586	<.0001*
Ecology	2	2	4.9617	28.2006	<.0001*
Log(Branch Length)	1	1	2343.0342	26633.97	<.0001*
Log(Time)	1	1	53.2527	605.3393	<.0001*
sign of beta	1	1	0.0368	0.4182	0.5179
Ecology (Aq, Te, Vo)*sign of beta	2	2	0.6607	3.7551	0.0235*

Note: significant F-test for the interaction accounts for <0.02% of the variance. Class codes four groups by including avian and non-avian Reptiles as one group. See Methods.

2b. Amphibians R^2 = 0.939

Source	No. parameters	DF	Sum of Squares	F Ratio	Prob > F
Ecology	1	1	0.36081	4.9231	0.0273*
Log(Branch Length)	1	1	250.35775	3416.038	<.0001*
Log(Time)	1	1	5.04316	68.8121	<.0001*
sign of beta	1	1	0.11483	1.5668	0.2117
Ecology *sign of beta	1	1	0.08887	1.2127	0.2717

2c. Birds R^2 = 0.937

Source	No. parameters	DF	Sum of Squares	F Ratio	Prob > F
Ecology	2	2	0.90325	5.5919	0.0040*
Log(Branch Length)	1	1	390.46049	4834.586	<.0001*
Log(Time)	1	1	13.21730	163.6534	<.0001*
sign of beta	1	1	0.13115	1.6239	0.2032
Ecology*sign of beta	2	2	0.20195	1.2503	0.2875

Note: a) there are only a small number of terrestrial and aquatic effects; b) sign of beta and its interaction with ecology are collinear. Removing the interaction the sign of beta effect can be estimated and is non-significant (F<1.0)

2d. Fish R^2 = 0.948

Source	No. parameters	DF	Sum of Squares	F Ratio	Prob > F
Ecology[a]	-	-	-	-	-
Log(Branch Length)	1	1	629.90321	7986.053	<.0001*
Log(Time)	1	1	10.17553	129.0077	<.0001*
sign of beta[b]	1	1	0.60266	7.6406	0.0058*
Ecology *sign of beta	0	0	0.00000	.	.

Note: a) there is no variation in Ecology within the Fish; b) the sign of beta effect accounts for less than 0.05% of the variance

2e. Mammals R^2 = 0.942

Source	No. parameters	DF	Sum of Squares	F Ratio	Prob > F
Ecology	2	2	2.31155	13.9887	<.0001*
Log(Branch Length)	1	1	506.92658	6135.487	<.0001*
Log(Time)	1	1	18.53498	224.3345	<.0001*
sign of beta	1	1	0.17274	2.0907	0.1490
Ecology*sign of beta	2	2	0.01573	0.0952	0.9092

2f. Reptiles R^2 = 0.926

Source	No. parameters	DF	Sum of Squares	F Ratio	Prob > F
Ecology	1	1	1.15258	10.9340	0.0010*
Log(Branch Length)	1	1	478.58606	4540.107	<.0001*
Log(Time)	1	1	10.04996	95.3390	<.0001*
sign of beta	1	1	0.00454	0.0430	0.8358
Ecology*sign of beta	1	1	0.00766	0.0727	0.7876

Supplementary Table 3. Marginal mean effects for Ecology

Group	R^2	**Aquatic**<br>marginal mean, n<br>95% CI<br>Pairwise differences	**Terrestrial**<br>marginal mean, n<br>95% CI<br>Pairwise differences	**Volant**<br>marginal mean,n<br>95% CI<br>Pairwise differences
Vertebrates n=2408<br>All p<0.0001	0.940	0.181, n=842<br>0.168 - 0.195<br>Te,Vo	0.137, n=1079<br>0.133 – 0.144<br>Aq,Vo	0.116, n=487<br>0.107 – 0.121<br>Aq,Te
Avian Reptiles (Birds), n=437	0.936	0.134, n=3<br>0.095 – 0.189	0.225, n=3<br>0.159-0.316<br>Vo	0.126, n=431<br>0.122 – 0.129<br>Te
Fish, n=806	0.948	0.213, n=806<br>0.208 – 0.216	n=0	n=0
Mammals, n=414<br>p<0.0005, Aq-Te p=0.003	0.942	0.204, n=13<br>0.172 – 0.241<br>Te,Vo	0.153, n=345<br>0.1480 – 0.158<br>Aq,Vo	0.123, n=56<br>0.113 – 0.137<br>Aq,Te
Non-Avian Reptiles, n=451<br>P<0.001	0.926	0.258, n=5<br>0.180 – 0.371<br>Te	0.140, n=446<br>0.136-0.145<br>Aq	n=0
Amphibians, n=300<br>p<0.025	0.939	0.124, n=15<br>0.107 – 0.143<br>Te	0.104, n=285<br>0.101 – 0.107<br>Aq	n=0

Note: The Vertebrate regression tests the effect of ecology controlling for Class, thermoregulation, branch length, time in the past, sign of the peak-shift and its interaction with ecology (see main text and Methods). Tests within a Class estimate the regression model separately within that Class so some results are based on small sample sizes. All pairwise differences between environments within a row are significant at the two-tailed level shown or less in the left column. All pairwise tests conducted using Tukey HSD criterion which simplifies to t-test when only two categories exist. The sample sizes refer to the number of peak-shifts in the particular category.

Supplementary Table 4 Marginal probabilities of change occurring in a branch[1].

Group<br>Pairwise p-values	Aquatic<br>marginal frequency, n	Terrestrial<br>marginal frequency, n	Volant<br>marginal frequency, n
Vertebrates, n=40749 branches<br><br>p<0.0001, Aq-Te p<0.025	0.081, n=10266<br>Te,Vo	0.062, n=16947<br>Aq,Vo	0.033,n=13536<br>Aq,Te
Avian Reptiles (Birds), n=12313 branches<br>Vo-Te p=0.073	0.104,*n=31*	0.082,n=25<br>Vo	0.039,n=12257<br>Te
Fish<br>n=9690 branches	0.071,n=9690	–	–
Mammals<br>n=6442 branches<br>p<0.0001, Aq-Te p=0.21	0.114,n=263<br>Vo	0.087,n=4900<br>Vo	0.046,n=1279<br>Aq,Te
Non-Avian Reptiles<br>n=7597 branches	0.035,n=117	0.053,n=7480	–
Amphibians<br>n=4707 branches<br>p<0.03	0.093,n=165<br>Te	0.056,n=4542<br>Aq	–

1) Marginal probabilities derived from logistic-regression models fitted to the Vertebrates as a whole and then individually within each Class, controlling for all background variables as in Supplementary Table 3; Notes: All pairwise differences between environments within a row are significant at the two-tailed level shown (odds-ratio test on the log(odds) controlling for multiple comparisons) or less in the left column, e.g., the Aquatic-vertebrates' marginal probability differs from the Vertebrate-Terrestrial and Volant probabilities. Non-Avian Reptiles' estimates derived excluding 9 branches in which the ecological state was uncertain between Te or Aq.

Within-population trait variances

Supplementary Table 5. Sources of Vertebrate within-population variances in size or size-related traits

Data	Web-link
Population characteristics of animal, plant, insect, crustacean and mollusc species, ref 1	https://www.annualreviews.org/doi/suppl/10.1146/annurev-ecolsys-011121-021241.
Population characteristics of animal, plant and insect species, ref 2,3	(https://datadryad.org/stash/dataset/doi:10.5061/dryad.7996).
Megachiroptera, ref 4	https://royalsocietypublishing.org/doi/full/10.1098/rspb.2025.0743#d1e1770
Cetacea, ref 5	https://doi.org/10.5519/vmbrpkuq.

We included only vertebrate species and excluded populations with CV>0.3, where CV is the coefficient of variation σ/μ in the raw data, and populations in reference [1] in which the additive genetic variance was estimated as equal to zero. Population variances were then converted to variance on a log-scale according to $\sigma^2(\ln(x)) \cong CV^2$ for CV≤0.3. In the cetacean and megachiropteran datasets the within-population variance increased with the number of individuals

sampled per species. We successively removed species with small sample sizes until the positive association vanished (<10 individuals sampled for megachiroptera, < 30 for cetacea) and then calculated variances on the remaining populations. Morphological traits in ref 1 were identified by the label “morph” in the category 1 variable and in ref 2 by the labels “mass” and “length” in the trait categories variable.

Scaling of β parameters associated with length measurements

To analyse the size changes across all five Vertebrate Classes simultaneously we rescaled those associated with body length data (Fish, Reptiles and Amphibians) according to the allometric relationship $mass \propto length^b$, where theory predicts that $b \cong 3$. On a log-scale the transformation is equivalent to multiplying the length measures by b. We set $b = 3$ based on literature sources (SI Table 6).

Supplementary Table 6. Allometric scaling exponents describing the relationship between body mass and length across three Vertebrate Classes

Class	Range of allometric scaling exponents	Average allometric scaling exponent	Reference
Fish	2.684 - 3.183	2.96	6
Fish	2.405 - 3.270	2.838	7
Fish	1.994 - 3.942	2.968	8
Fish	2.334-3.564	2.949	9
Reptiles (snakes)	2.340 - 3.631	2.986	10
Reptiles (lizards)	-	2.913	11
Reptiles (lizards)	3.040 - 3.165	3.108	12
Reptiles (lizards)	2.930 - 3.220	3.075	13
Amphibians (frogs)	-	3.088	14
Amphibians (salamanders)	-	2.895	15
Amphibians (frogs)	-	3.005	16

Priors on model parameters

SI Table 7 reports the prior distributions used in the Bayesian Markov chains. For the ancestral body size (also known as the root and denoted by the parameter, α) we investigated the natural history of each Vertebrate class to centre the prior on alpha around the mass or length of a fossil near the root date of each tree. For mammals, we used the estimated body mass of the fossil *Eomaia scansoria* (~25g; ref 11). For the Birds' alpha prior we used an estimated body mass of 450g for the fossil *Archaeopteryx lithographica* (ref 17) as the prior's mean and for the Fish, we calculated the mean length of a sample of Devonian Actinopterygian fossils (25mm; ref 18). Owing to a lack of early, post-cranial fossils we derived priors centred on the midpoint length of the extant taxa for both Reptiles and Amphibians. The remaining priors are from our earlier work (ref 19). Some ranges when converted to grams or millimetres are unrealistic but the likelihood is calculated in log-space.

Supplementary Table 7. Prior distributions of model parameters by Vertebrate Class

Parameter<br><br>Prior distribution	Alpha (α)<br><br>Gamma	Mean (95% CI) body size of prior distribution on Alpha	Brownian variance (σ^2) Uniform	Betas (β)<br><br>Weibull	variance scalars (υ)<br><br>Gamma	Global beta<br><br>Normal
Mammals	α=2, β=0.7	25g<br>(1.8 – 2300 g)	0 - 1	λ=1.1, κ=1.5	α=1.2, β=5	μ=0, σ=0.1
Birds	α=10, β=0.265	450g<br>(25 – 12500 g)	0 - 1	λ=1.1, κ=1.5	α=1.2, β=5	μ=0, σ=0.1
Reptiles	α=2, β=1.85	5000mm<br>(4 – 108 mm)	0 - 1	λ=0.6, κ=1.4	α=1.2, β=5	μ=0, σ=0.1
Amphibians	α=2, β=1.42	691mm<br>(1.6 – 3.9 x 10^6 mm)	0 - 1	λ=0.6, κ= 1.4	α=1.2, β=5	μ=0, σ=0.1
Fish	α=2, β=0.7	25mm<br>(1.8 – 2300 mm)	0 - 1	λ=0.6, κ=1.4	α=1.2, β=5	μ=0, σ=0.1

The variance-covariance matrix of the peak-shift (β) parameters

The multiple linear regression and other analyses we perform on the βs require the standard assumption that the variance-covariance (VC) matrix of the errors of prediction is diagonal and homoscedastic. We calculated the variance-covariance matrix of the βs taking it as an indicator of the error matrix, by sampling βs at widely-spaced intervals from the stationary Markov chains and then calculated the $n(n-1)/2$ unique pairwise correlations among them, where n is the number of βs. For mammals, for example, the 414 βs yield 85,491 unique pairwise correlations. We repeated this for each Class (SI Table 8).

Overall 98.5% of the correlations do not differ significantly from zero at the α =0.05 level after controlling for Type I errors using the Hochberg FDR correction; the more conservative Bonferroni correction yields 99.6% non-significant. The geometric mean coefficient of variation of the βs is 0.019 (median = 0.019, IQR = 0.012-0.029), and their variances are only negligibly correlated with their means (r^2 = 0.0003; see SI Table 8 for results within Classes). Combined with the pairwise correlations this is evidence for homoscedaticty of the (small) variances and of βs that are

independent of one another. It is reasonable to suppose from this that the variance-covariance matrix of the errors of the regressions are diagonal matrices with roughly equal variances along their main diagonals.

Supplementary Table 8 Statistics on the variance-covariance matrix of the βs

Class	No. of βs	Percent pairwise correlations not significant, Hochberg FDR	Geometric mean coefficient of variation for β	r^2 (mean and variance of β)
Amphibians	300	99.3	0.016	0.009
Birds	437	98.2	0.019	0.007
Fish	806	98.1	0.021	0.0005
Mammals	414	99.1	0.020	0.0008
Reptiles	451	97.7	0.017	0.0005

Supplementary Information References

1. T. F. Hansen, C. Pélabon, Evolvability: A Quantitative-Genetics Perspective. *Annual Review of Ecology, Evolution, and Systematics* **52**, 153-175 (2021).
2. J. G. Kingsolver *et al.*, The strength of phenotypic selection in natural populations. *The American Naturalist* **157**, 245-261 (2001).
3. J. G. Kingsolver, S. E. Diamond, Phenotypic selection in natural populations: what limits directional selection? *The American Naturalist* **177**, 346-357 (2011).
4. Almeida, C., Francisca, Helgen, K. M., Simmons, N. B. & Giannini, N. P. Evolution and ecology of body size in the world's largest bats. *Proceedings B* **292**, 20250743 (2025).
5. Burin, G., Park, T., James, T. D., Slater, G. J. & Cooper, N. The dynamic adaptive landscape of cetacean body size. *Current Biology* **33**, 1787-1794. e1783 (2023).
6. Riedel, R., Caskey, L.M. and Hurlbert, S.H., 2007. Length-weight relations and growth rates of dominant fishes of the Salton Sea: implications for predation by fish-eating birds. *Lake and Reservoir Management*, *23*(5), pp.528-535.
7. Mehanna, S.F. and Farouk, A.E., 2021. Length-Weight Relationship of 60 Fish Species From the Eastern Mediterranean Sea, Egypt (GFCM-GSA 26). *Frontiers in Marine Science*, *8*, p.625422.
8. Kulbicki, M., Mou Tham, G., Thollot, P. and Wantiez, L., 1993. Length-weight relationships of fish from the lagoon of New Caledonia. *Naga Iclarm Q*, *16*(2-3), pp.26-29.
9. Sangun, L., Akamca, E. and Akar, M., 2007. Weight-length relationships for 39 fish species from the north-eastern Mediterranean coast of Turkey. *Turkish Journal of Fisheries and Aquatic Sciences*, *7*(1).
10. Feldman, A. and Meiri, S., 2013. Length–mass allometry in snakes. *Biological Journal of the Linnean Society*, *108*(1), pp.161-172.
11. Meiri, S., 2010. Length–weight allometries in lizards. *Journal of Zoology*, *281*(3), pp.218-226.
12. Vitt, L.J., van Loben Sels, R.C. and Ohmart, R.D., 1981. Ecological relationships among arboreal desert lizards. *Ecology*, *62*(2), pp.398-410.
13. Vitt, L.J. and Pianka, E.R., 2004. Historical patterns in lizard ecology: what teiids can tell us about lacertids. *The Biology of Lacertid lizards: Evolutionary and ecological perspectives*, *8*, pp.139-157
14. Santini, L., BENÍTEZ-LÓPEZ, A., Ficetola, G.F. and Huijbregts, M.A., 2018. Length–mass allometries in amphibians. *Integrative zoology*, *13*(1), pp.36-45.

15. Landler, L., Burgstaller, S., Spießberger, M., Horvath, A., Zhelev, Z., Mollov, I., Sinsch, U., Nepita, J., Schwabel, F., Kuhn, W. and Köbele, C., 2022. A Unified Approach to Analysis of Body Condition in Green Toads. *Diversity*, *15*(1), p.43.
16. Ji, Q., Luo, Z.X., Yuan, C.X., Wible, J.R., Zhang, J.P. and Georgi, J.A., 2002. The earliest known eutherian mammal. *Nature*, *416*(6883), pp.816-822.
17. Serrano, F.J., Palmqvist, P. and Sanz, J.L., 2015. Multivariate analysis of neognath skeletal measurements: implications for body mass estimation in Mesozoic birds. *Zoological Journal of the Linnean Society*, *173*(4), pp.929-955.
18. Albert, J.S., Johnson, D.M. and Knouft, J.H., 2009. Fossils provide better estimates of ancestral body size than do extant taxa in fishes. *Acta Zoologica*, *90*, pp.357-384.
19. Pagel, M, O'Donovan, C and Meade, A. 2022. General statistical model shows that macroevolutionary patterns and processes are consistent with Darwinian gradualism. *Nature communications* **13**, pp.1-12.
