# Supplementary material for: Convergence in the size and frequency of macroevolutionary peak-shifts on the adaptive landscape in vertebrates
Source: Nat Commun. 2026 Oct 27;17:11208. doi: 10.1038/s41467-026-78289-z (PMC13720082; doi:10.1038/s41467-026-78289-z)
Supplement: Supplementary file 2 [file 41467_2026_78289_MOESM2_ESM.pdf]

nature portfolio

Peer Review File

Convergence in the size and frequency of macroevolutionary peak-shifts on the adaptive landscape in Vertebrates

Corresponding Author: Professor Mark Pagel

This file contains all reviewer reports in order by version, followed by all author rebuttals in order by version.

Version 0:

Reviewer comments:

Reviewer #1

(Remarks to the Author)
This interesting manuscript reports on an analysis of body size evolution in five of the major radiations of vertebrates (amphibians, lepidosaurs, ray-finned fishes, mammals, and birds). The study focuses on the results of implementation of the 'fabric model' that was introduced in 2022 by the same group of authors. They find a tendency for the patterns of peak shifts to be most dynamic in aquatic habitats where shifts are larger and more frequent than terrestrial habitats and smallest and least frequent in volant lineages. This is where the term 'convergence' in the manuscript's title derives from. The manuscript is mostly clearly written and easy to follow and overall I found it an impressive implementation of the authors' new model. The model output is carefully analyzed with various regressions. However, there are a number of issues with the manuscript that I found either awkward or problematic – and, forgive me, but I will focus on those in my comments below.

1. On the one hand the results can be seen as unsurprising in the sense that we already know a lot about the relationship between body size and major habitats in vertebrates. Yes, marine mammals tend to be larger than terrestrial and flying mammals, and flying vertebrates are the smallest. The authors could do more to use their results to derive novel biological insights into vertebrate diversification and articulate these insights. I do not mean that I find the results of their study to be trivial, I am saying that there is room for improvement in the description of any novel insights into vertebrate evolution. I fear the authors will be put off by this comment and be tempted to wave it off but please give it some careful thought. I think it could significantly affect the impact of the paper.

2. It would be helpful to have another go at the first couple of paragraphs of the introduction. The first sentence appears to define macroevolution as the evolution of major differences among species. That does not seem to be an accurate characterization of macroevolution and I'm not even sure I've ever heard anyone describe the evolution of major differences among species as 'macroevolution' (until reading this manuscript).

3. Similarly, I'm not so sure about the ubiquity of stasis and punctuation mentioned in the second sentence. That is one pattern that is observed but how widespread it is seems still up for debate.

4. The third paragraph of the introduction introduces some ideas around the evolutionary lines of least resistance – or evolution being influenced by patterns of genetic variance-covariance structure among traits. It might be better to focus here on the importance of genetic variance for a single trait, since this paper deals with only one trait (body size).

5. A concern about the model fitting exercise is that the fabric model is only compared to two rather unlikely alternatives (Table S1). As these authors have themselves previously pointed out – models of trait evolution tend to find the things that they were designed to find. The comparison of the current model to simple Brownian motion and white-noise seems like a weak confirmation that the model is an improvement over a multi-peak OU model or a Levi process model. If more appropriate models aren't compared then some rationale for that decision should be added.

6. Given that the title emphasizes convergent evolution, I recommend the authors more carefully flesh out which patterns are seen in multiple lineages and which are not. The possibility of convergence should be addressed more rigorously, rather than the informal way is treated now.

7. The ms focuses on t he use of a model introduced a few years ago by these authors. Considering that readers will likely

not be familiar with the model, an effort should be made to clearly explain the model parameters and how it is different from related models that are more familiar (such as OU models). This is not given enough attention in this manuscript and one needs to go back to the 2022 paper to round out understanding how this model works.

8. A minor point about the text is that many words are unnecessarily capitalized. This is not a big deal and I realize that conventions may vary on something like this. The most common example is the word ‘vertebrates’ which is capitalized throughout but many other words as well: mammals, birds, amphibians, fish. Perhaps this is a British thing? Taxonomic names are capitalized (e.g. Amphibia, Mammalia) but not when referring to mammals or amphibians, etc.

9. Page 6. This survey of within population variance in traits. Growth patterns differ markedly among vertebrate classes. In particular, fishes have much more indeterminate growth than mammals or birds. My guess is that this results in much greater within population variation in body size of fishes.

(Remarks on code availability)

Reviewer #2

(Remarks to the Author)
In this manuscript, the authors investigated the frequency and magnitude of shifts in trait evolution across the major clades of vertebrates to ask (1) whether a Simpsonian model of evolution on a rugged adaptive landscape is supported, and (2) whether microevolutionary constraints can explain detected macroevolutionary patterns. The authors employed their recent "fabric" model of macroevolutionary change to do this. They also tested for emergent ecological rules in the magnitude and frequency of large evolutionary changes in these clades. The authors found support for a rugged model of the adaptive landscape, and identified consistent differences between major ecological groups (aquatic, terrestrial, aerial) in the nature of evolutionary change, and in the amount of population-level trait variation.

I enjoyed this manuscript. The authors used their recently-developed approach to detect novel and interesting evolutionary patterns out of a large dataset on vertebrate body size. Their interpretation of results is biologically sensible, and they make a compelling case for a potential link between microevolutionary processes and macroevolutionary patterns. While I can imagine that some may favor other macroevolutionary modeling frameworks over the fabric approach (e.g., because the latter is very parameter rich), I believe it is at least as sensible an approach as other commonly-used phenomenological models of macroevolution. I think this paper will advance the ongoing conversation about the tempo and mode of macroevolution.

I do have a few criticisms, concerns, or suggestions about this work, but these are issues that I think should be manageable in a revision. Perhaps my main comments concern the phylogenetic data used in the study, and some potential ambiguity about how constraint is discussed in the manuscript.

Regarding the phylogenetic data, my biggest worry with studies that use heterogenously-assembled supertrees (like those in TimeTree) is the nature of the branch lengths. In this study, I noted the existence of polytomies, which I strongly suspect reflect a lack of information about the timing of divergence for such groups. Such issues would probably be expected to have a bearing on the estimation of parameters under the Fabric model. How much does this matter? For starters, it would be useful to know how well resolved the TimeTree phylogeny is. E.g., what percentage of branches originate from polytomies in the final utilized tree, and does that vary by clade or ecology? If so, could this potentially lead to spurious results? Also, I would be curious to see how results compare when using a carefully time-calibrated published tree for a well-studied group (e.g., some well-studied subclade) versus a tree for that same group, but obtained from the TimeTree web resource. There should be several sizeable published timetrees that could be utilized for this (e.g., various mammal trees from the Slater lab).

All of my other comments refer to specific sections of text. I've organized them by paragraph, as there were no line numbers in my copy of the manuscript:

Main, Paragraph 3: some may take issue with the term 'equivalently' when used to relate genetic lines of least resistance to limitations imposed by mutational variance. The G matrix reflects both the mutational matrix M and the history of selection on those incoming mutations, so the genetic constraints it imposes are not strictly due to mutation. Indeed, the last paragraph of Main implies that within-population variance reflects the strength of stabilizing selection, rather than mutational variance.

Results, Paragraph 10: I was surprised how few peak shifts there were for terrestrial birds (n=1). Is that because there were few terrestrial birds in the tree, or because they didn't exhibit shifts? I would have expected lots of options, as there are many flightless birds out there.

Results, Paragraph 12: I'm skeptical of the accuracy of the Reptile aquatic shift frequency result, given that most aquatic reptiles are now extinct (e.g., plesiosaurs, pliosaurs, mosasaurs, countless extinct crocodilians and turtles). Many of these were very large, and I suspect these extinct lineages would have provided evidence for more frequent (and even higher magnitude) shifts. Perhaps this is worth a speculative mention.

Results, Paragraph 13: I was a little confused by the discussion here about whether terrestrial and aerial changes are

constrained (or aquatic ones unconstrained), and specifically the suggestion at the end of the paragraph that constraints likely aren't the reason for the differences in sizes of peak shifts. What is meant here by 'constrained'? My initial interpretation was that you must be talking about selective or functional constraints (in the terminology of Arnold 1992, Am. Nat.), as that would be appropriate for a constraint imposed by an external factor such as buoyancy. However, in explaining why you don't think constraints explain the patterns (in the next two paragraphs), the explanation given is mutational, which is a different type of evolutionary constraint (i.e., this would be Arnold's 'genetic' constraint). Such intrinsic constraints are different from extrinsic constraints such as neutral buoyancy. I think some clarification is needed here. It seems to me that the analyses here are equipped to evaluate the role of mutational constraint, but I'm not sure whether the same can be said for extrinsic constraints.

Discussion, Paragraph 4-7: I was a little surprised not to see any discussion of the vastly different requirements for supporting body weight against gravity as a reason for the differences in the adaptive landscapes in water, on land, and in air. I agree that different circulatory needs are probably at play, and that aquatic environments probably overwhelming select for fusiform shapes, but supporting the body is probably a key factor here. Water makes it much easier to evolve from small to large, and vice versa, because it buffers the structural support needs of vertebrates (beached whales illustrate this well). The aerodynamic requirements of powered flight work in the other direction, making body size evolution a greater task than in terrestrial relatives, due to the need to ensure that size evolution is accompanied by the concomitant shape evolution necessary to keep the animal airborne.

Discussion, Paragraph 5: Here it is stated that aquatic reptiles have small magnitudes of peak shifts, but Figure 2 suggests this was an error (aquatic reptiles have the largest shift magnitude of all). As noted earlier, I suspect this magnitude is probably an underestimate, given that extant phylogenies poorly represent the full size range of marine reptiles throughout their history.

Methods, Statistical Analyses, Paragraph 2: are the betas indeed actually phylogenetically controlled? Even though they were obtained using a phylogenetic comparative method, might they not still exhibit phylogenetic autocorrelation that should be accounted for, if present?

Methods, Stat Analyses, Paragraph 3: "since the present" should be "before the present"

(Remarks on code availability)

Version 1:

Reviewer comments:

Reviewer #1

(Remarks to the Author)
This is a very satisfying revision of a manuscript I reviewed earlier. I was previously concerned that the paper would be hard to access for people unfamiliar with the fabric model or comparative analyses in general. I feel the authors have elaborated enough on numerous points that the paper will reach a broader audience now. Also, the case for the biological significance of peak-shift size and results about this parameter are clearer. I thank the authors for tolerating my earlier questions and making these changes. I remain interested in how this approach will play out when more complex multivariate data sets are analyzed in a similar way, but I don't think that needs to be dealt with here. This is a very satisfying first step and body size is a compelling univariate trait. I am happy with this ms as it is and congratulate the authors.

Peter Wainwright

(Remarks on code availability)
no comments

Reviewer #2

(Remarks to the Author)
I was reviewer 2 for the original manuscript.
The authors have prepared a revision, and I think this revised manuscript nicely addresses the issues raised. I have just a few additional comments.

First, in the abstract, it would be helpful to clarify that you infer the population-level variance patterns to arise from evolution on a macroevolutionary adaptive landscape. Otherwise I think the vast majority of readers (including many who will only read the abstract) will assume that your claimed link between micro and macroevolution refers to the traditional concept of intrinsically constrained evolution along genetic lines of least resistance.

Lines 98-102: I found the text about Brownian motion and optima confusing here. The text describes "a Brownian diffusion process (an unbiased random-walk)
augmented by abrupt shifts in the position of optima on the adaptive landscape".

Do you also mean the abrupt appearance of an offset optimum? If there optimum were already present, how could evolution be Brownian?

343-345 - These lines seem to imply that peak shifts are associated with speciation events (if speciation is decoupled from shifts, the maintenance of genetic identities in lineages occupying different peaks is much less of a concern). Also, the dominance of allopatry as a speciation mode in vertebrates should largely resolve this concern.

Finally, for the regression of estimated betas:
While I appreciate that beta effects are not expected to be accumulated up the tree, I'm not convinced that this completely rules out the possibility that there could be phylogenetically correlated errors in a regression of them on the proposed independent variables. My remaining concern stems from the fact that these betas are not data, but rather are parameter estimates arising from a fitted model. There will be error associated with their estimation (variances), but because they are jointly estimated, there will also be joint error (covariances) associated with their estimation. Is it not possible that such covariances might reflect the phylogeny?
Ultimately I view this as an empirical matter. While I don't have any particularly strong reason to suspect strongly phylogenetically correlated errors for the betas, it seems like something that could be tested, and given that this is a relatively new framework that others will adopt, I think there would be value in looking into this.

(Remarks on code availability)

Open Access This Peer Review File is licensed under a Creative Commons Attribution 4.0 International License, which permits use, sharing, adaptation, distribution and reproduction in any medium or format, as long as you give appropriate credit to the original author(s) and the source, provide a link to the Creative Commons license, and indicate if changes were made.
In cases where reviewers are anonymous, credit should be given to 'Anonymous Referee' and the source.
The images or other third party material in this Peer Review File are included in the article's Creative Commons license, unless indicated otherwise in a credit line to the material. If material is not included in the article's Creative Commons license and your intended use is not permitted by statutory regulation or exceeds the permitted use, you will need to obtain permission directly from the copyright holder.
To view a copy of this license, visit https://creativecommons.org/licenses/by/4.0/

RESPONSE TO REVIEWERS' COMMENTS

We thank both reviewers for their thoughtful comments. Please see replies below in italics.

Reviewer #1 (Remarks to the Author):

This interesting manuscript reports on an analysis of body size evolution in five of the major radiations of vertebrates (amphibians, lepidosaurs, ray-finned fishes, mammals, and birds). The study focuses on the results of implementation of the 'fabric model' that was introduced in 2022 by the same group of authors. They find a tendency for the patterns of peak shifts to be most dynamic in aquatic habitats where shifts are larger and more frequent than terrestrial habitats and smallest and least frequent in volant lineages. This is where the term 'convergence' in the manuscript's title derives from. The manuscript is mostly clearly written and easy to follow and overall I found it an impressive implementation of the authors' new model. The model output is carefully analyzed with various regressions. However, there are a number of issues with the manuscript that I found either awkward or problematic – and, forgive me, but I will focus on those in my comments below.

1. On the one hand the results can be seen as unsurprising in the sense that we already know a lot about the relationship between body size and major habitats in vertebrates. Yes, marine mammals tend to be larger than terrestrial and flying mammals, and flying vertebrates are the smallest. The authors could do more to use their results to derive novel biological insights into vertebrate diversification and articulate these insights. I do not mean that I find the results of their study to be trivial, I am saying that there is room for improvement in the description of any novel insights into vertebrate evolution. I fear the authors will be put off by this comment and be tempted to wave it off but please give it some careful thought. I think it could significantly affect the impact of the paper.

If we understand the reviewer we don't think our results are about size per se ("marine mammals tend to be larger than terrestrial and flying mammals *…."). Fish (generally small in size) have slightly larger peak shifts on average than those in the aquatic mammals (generally very large in size). Many terrestrial species (with intermediate-sized peak shifts) are larger in size than many fish, and some birds (with very small peak shifts) are larger than many of the smaller ectothermic terrestrial species (lizards, frogs).*

Instead, we think the results say something about how the fitness landscapes of aquatic, terrestrial and aerial environments affect the size and frequency of peak shifts. We've gone back through the paper and made several revisions to emphasise these points. Please see especially lines 333-348 in the Discussion. Aquatic environments (we suggest) flatten the fitness curve for size. Aerial environments, we suggest, do the reverse, keeping a tight rein on variation within a species in size. Terrestrial environments fall somewhere in the middle. The within-population variance data support this rank-ordering.

This structuring of the adaptive landscape then determines how far apart new macroevolutionary optima must be for species to maintain their genetic identities: aquatic species have to move further, aerial species less. This then translates into the differing macroevolutionary patterns we observe: fish and cetacea, seals, sea lions and penguins have to change more when moving to new optima not because it is 'easier' to get big in water (peak shifts measure increases and decreases in size) but because neutral buoyancy in water means that the fitness curve for size within a species is relaxed, and vice versa for aerial species. Yes, some aquatic mammals are huge and this is down to neutral buoyancy, but they didn't need a succession of large macroevolutionary peak shifts to become huge.

2. It would be helpful to have another go at the first couple of paragraphs of the introduction. The

first sentence appears to define macroevolution as the evolution of major differences among species. That does not seem to be an accurate characterization of macroevolution and I'm not even sure I've ever heard anyone describe the evolution of major differences among species as 'macroevolution' (until reading this manuscript).

We've changed our first sentence to "Evolutionary biologists use the term macroevolution to refer to the processes that lead to differences among species and higher taxonomic groups as opposed to microevolution which refers to evolution that occurs within a species". Lines 41-43.

This is similar to a number of authors. For example, our original sentence is close to Arnold's who defines macroevolution as the "processes responsible for the divergence among genera or higher taxa", Arnold, Pfrender and Jones, The adaptive landscape as a conceptual bridge between micro- and macroevolution. Genetica, 2001, Volume 112, pages 9–32; we are not that far off Jablonski's definition of macroevolution as "Macroevolution, defined broadly as evolution above the species level….". Evol Biol, 2017 Jun 3;44(4):427–450. doi: 10.1007/s11692-017-9420-0; or from The Stanford Encyclopedia of Philosophy Archive (2009) which defines macroevolution as "Macroevolution refers (most of the time, in practice) to evolutionary patterns and processes above the species level".

Other authors go further (Schluter, Ricklefs) but we don't think more detail or elaboration is needed here.

3. Similarly, I'm not so sure about the ubiquity of stasis and punctuation mentioned in the second sentence. That is one pattern that is observed but how widespread it is seems still up for debate.

*We've changed that sentence to read "One of this field's most enduring observations is that morphological change over evolutionary timescales – as witnessed in the fossil record and phylogenetic datasets – can **often** be characterised by long periods of stasis during which little or no change occurs, but interspersed with shorter bouts of abrupt, episodic or punctuational change[references]". Please see line 45.*

We think this is justified. We have reported abrupt or punctuational-like change in data ranging from genes to languages, consistent with reports from others. In phenotypic data such as here we generally find that there is only a very weak association between the amount of change in a branch and the length of that branch in units of time: short branches tend to show approximately the same overall change as longer ones (please see the section in our paper about this (Magnitude of peak shifts is only weakly related to time). This is the pattern expected under Lande's 1985,1986 models of punctuational change – species spend most of their time on adaptive peaks with occasional shifts between peaks that take comparatively little time. It's also consistent with Uyeda et al's work (PNAS, The million-year wait) on the "blunderbuss" pattern.

4. The third paragraph of the introduction introduces some ideas around the evolutionary lines of least resistance – or evolution being influenced by patterns of genetic variance-covariance structure among traits. It might be better to focus here on the importance of genetic variance for a single trait, since this paper deals with only one trait (body size).

We prefer to retain the context of lines of least resistance, but we now add 5 lines at the start of the following paragraph regarding the evolution of a single trait. Please see lines 81-85. We wish to keep this context because it prompted debates about whether macroevolution was following Gmax versus Gmax being sculpted by species' responses to selection. We return to this debate in the Discussion where we make the case based on our data that environments sculpt genetic variation rather than the pre-existing genetic variation setting the trajectory of macroevolution.

5. A concern about the model fitting exercise is that the fabric model is only compared to two rather unlikely alternatives (Table S1). As these authors have themselves previously pointed out – models of trait evolution tend to find the things that they were designed to find. The comparison of the current model to simple Brownian motion and white-noise seems like a weak confirmation that the model is an improvement over a multi-peak OU model or a Levi process model. If more appropriate models aren't compared then some rationale for that decision should be added.

We've revised the manuscript to explain more fully our choice of the Fabric model (see lines 98-121), including two key ways it differs from OU models and from Levy process models. The Fabric model, unlike these other models, does not link the size of peak shift changes to variance changes and we've added a remark early in the Results that shows there is no relationship between the two in our data (lines 144-148). The Fabric model also presumes species adapt to new optima effectively instantly in macroevolutionary time, and we provide evidence in the Results on how small the typical peak shifts are (section on Macroevolutionary peak shifts are small).

We've added a sentence to the results about the white-noise version of the Fabric model saying that the improvement of the default Fabric model over its white-noise version is consistent with the view that species are tracking continually moving adaptive peaks. Please see lines 139-139.

Beyond these two points we do not wish this paper to be about comparisons among models, especially given the growing evidence that OU models can be difficult to fit (largely because of the way the model links α and σ^2, see for example Ho and Ané 2013, 2014 on identifiability, or Uyeda and Harmon's own discussion of this topic on "likelihood ridges" and failures of convergence) and that they underestimate the number of peak shifts.

Uyeda and Harmon in their paper on the multi-peak OU report a median marginal likelihood of -143.6 for their model applied to Chelonian carapace data, and they find 16 peak shifts. We fit the Fabric model to the same data using their priors for the variance and the root value (the parameters the models have in common) and otherwise our usual lenient priors. We find a substantially improved median marginal likelihood of -73.6 (or average of -74.7±8.7, based on 18 independent MCMC chains) and we find a median (and mean) of 25 peak shifts.

Uyeda and Harmon find a "half-life" of around 4 million years for the Chelonian carapace data. Assuming a thirty to forty-year generation time for these turtles, that is around 100,000 generations. Even at a rate of 0.001 SDs of change per generation these turtles could produce around 100 SDs of change in that time. The peak shifts we and they identify are orders of magnitude smaller than 100 SDs.

Similarly, Burin et al (Curr Biol. 2023) in their analyses of cetaceans remark at how few peak shifts the multi-peak OU model finds. They fit the Fabric model and then report that "we identified 18 branches along which evolution was biased away from an expected change of 0, none of which were found by bayou" [bayou is the software that implements the multi-peak OU]. They later "argue that the close proximity of these long-term means [the optima] facilitates movement between peaks on the adaptive landscape in relatively small increments, which are not identified as discrete shifts in our bayou analyses but appear as trends in the analysis using the Fabric model". Burin et al find a half-life estimate of 90 million years for the cetacean data. Such an excessively long half-life(about 1.5 times the age of the clade) points to the cetacean data as being not far off a Brownian process.

6. Given that the title emphasizes convergent evolution, I recommend the authors more carefully flesh out which patterns are seen in multiple lineages and which are not. The possibility of convergence should be addressed more rigorously, rather than the informal way is treated now.

We now include a table in the main text (Table 1) that identifies 95 independent instances of peak shifts in single species or lineages (encompassing 304 species) that occupy an ecology that differs from their Class's ancestral ecology (e.g., bats). Call these 'derived ecology species'. We show that the marginal mean peak shift magnitudes of these 95 derived ecology cases in the three ecological categories follow the same rank-order and have similar magnitudes to the size of peak shift magnitudes of species that have never moved from the ecology that is basal to their Class (call these 'ancestral ecology species'). And they both resemble the Vertebrates as a whole. We provide a table of Supplementary data listing all the derived ecology species. We also pick out several species and lineages for mention in the main text. Please see ines 218-228

We repeat this exercise for the probability of a peak shift occurring in a branch (Table 2). Please see lines 248-259.

7. The ms focuses on the use of a model introduced a few years ago by these authors. Considering that readers will likely not be familiar with the model, an effort should be made to clearly explain the model parameters and how it is different from related models that are more familiar (such as OU models). This is not given enough attention in this manuscript and one needs to go back to the 2022 paper to round out understanding how this model works.

As above, we've revised the manuscript to explain more fully our choice of the Fabric model (see lines 98-121). Much of this draws on our 2022 paper that introduced that model including how it differs from OU and other models. That paper is open access and has additional treatment of how the model differs from OU, Levy and other models.

8. A minor point about the text is that many words are unnecessarily capitalized. This is not a big deal and I realize that conventions may vary on something like this. The most common example is the word 'vertebrates' which is capitalized throughout but many other words as well: mammals, birds, amphibians, fish. Perhaps this is a British thing? Taxonomic names are capitalized (e.g. Amphibia, Mammalia) but not when referring to mammals or amphibians, etc.

We've removed the unnecessary capitalisation throughout, restricting the use of capitals to when we use these terms as proper nouns as in "the XXXX", or implicitly so, or in column/row headings in Tables or in captions.

9. Page 6. This survey of within population variance in traits. Growth patterns differ markedly among vertebrate classes. In particular, fishes have much more indeterminate growth than mammals or birds. My guess is that this results in much greater within population variation in body size of fishes.

We now mentioned this. Please see lines 289-291.

Reviewer #2 (Remarks to the Author):

In this manuscript, the authors investigated the frequency and magnitude of shifts in trait evolution across the major clades of vertebrates to ask (1) whether a Simpsonian model of evolution on a rugged adaptive landscape is supported, and (2) whether microevolutionary constraints can explain detected macroevolutionary patterns. The authors employed their recent "fabric" model of macroevolutionary change to do this. They also tested for emergent ecological rules in the magnitude and frequency of large evolutionary changes in these clades. The authors found support

for a rugged model of the adaptive landscape, and identified consistent differences between major ecological groups (aquatic, terrestrial, aerial) in the nature of evolutionary change, and in the amount of population-level trait variation.

I enjoyed this manuscript. The authors used their recently-developed approach to detect novel and interesting evolutionary patterns out of a large dataset on vertebrate body size. Their interpretation of results is biologically sensible, and they make a compelling case for a potential link between microevolutionary processes and macroevolutionary patterns. While I can imagine that some may favor other macroevolutionary modeling frameworks over the fabric approach (e.g., because the latter is very parameter rich), I believe it is at least as sensible an approach as other commonly-used phenomenological models of macroevolution. I think this paper will advance the ongoing conversation about the tempo and mode of macroevolution.

I do have a few criticisms, concerns, or suggestions about this work, but these are issues that I think should be manageable in a revision. Perhaps my main comments concern the phylogenetic data used in the study, and some potential ambiguity about how constraint is discussed in the manuscript.

Regarding the phylogenetic data, my biggest worry with studies that use heterogenously-assembled supertrees (like those in TimeTree) is the nature of the branch lengths. In this study, I noted the existence of polytomies, which I strongly suspect reflect a lack of information about the timing of divergence for such groups. Such issues would probably be expected to have a bearing on the estimation of parameters under the Fabric model. How much does this matter? For starters, it would be useful to know how well resolved the TimeTree phylogeny is. E.g., what percentage of branches originate from polytomies in the final utilized tree, and does that vary by clade or ecology? If so, could this potentially lead to spurious results? Also, I would be curious to see how results compare when using a carefully time-calibrated published tree for a well-studied group (e.g., some well-studied subclade) versus a tree for that same group, but obtained from the TimeTree web resource. There should be several sizeable published timetrees that could be utilized for this (e.g., various mammal trees from the Slater lab).

We don't think that polytomies are playing a confounding role (see Table next page). The TimeTree varies between about 4 and 9% polytomies (nodes of >2 descendants) by Class (see Table below). Peak shifts are significantly more likely in polytomies than in bifurcating nodes in two of the five Classes (bottom row). But there is no relationship between the proportion of nodes that are polytomies and the rates at which we find peak shifts: for example, fish have the highest proportion of polytomies and a high rate of peak shifts overall but volant versus aquatic birds have similar and relatively high proportion of polytomies but the lowest and highest rates of peak shifts respectively. The same for mammals: their proportion of polytomies is similar for aquatic and volant forms but with very different rates of peak shifts. Mammals' aquatic peak shifts occur at similar rates to those of fish but with different levels of polytomies. The number of descendants from a node is not significant when added to the logistic regressions, either overall or separately within Classes.

We also don't think that branch lengths or timings in the past play much of a role. The estimates of the betas are sensitive to the total amount of change in a branch, and this is only very loosely related to the length of the branch. We also control for time in the past and branch length in our regression and logistic analyses.

Proportion of nodes with 2 or more descendants that are polytomies, by Class and Ecology. Polytomy defined as a node with >2 descendant branches

	Amphibians	*Birds*	*Fish*	*Mammals*	*Reptiles*
Total No. of nodes of 2 or more species	2272	5909	4471	3043	3680
proportion of nodes that are polytomies	0.045±0.004	0.062±0.003	0.093±0.004	0.073±0.005	0.044±0.003
Mean number of branches per node	2.07±0.39	2.08±0.38	2.17±0.71	2.12±0.59	2.06±0.36
Aq proportion polytomy	0	0.07	0.093	0.052	0.021
Te proportion polytomy	0.046	0	–	0.079	0.044
Vo proportion polytomy	–	0.062	–	0.055	-
Probability of a peak shift per branch in polytomy vs bifurcating node	0.073/0.063 p>0.25	0.053/0.033 P<0.01	0.085/0.083 p>0.35	0.083/0.061 P<0.03	0.072/0.058 p>0.25

All of my other comments refer to specific sections of text. I've organized them by paragraph, as there were no line numbers in my copy of the manuscript:

Main, Paragraph 3: some may take issue with the term 'equivalently' when used to relate genetic lines of least resistance to limitations imposed by mutational variance. The G matrix reflects both the mutational matrix M and the history of selection on those incoming mutations, so the genetic constraints it imposes are not strictly due to mutation. Indeed, the last paragraph of Main implies that within-population variance reflects the strength of stabilizing selection, rather than mutational variance.

That was poor phrasing. We've revised this paragraph…..Please see lines 64-65.

Results, Paragraph 10: I was surprised how few peak shifts there were for terrestrial birds (n=1). Is that because there were few terrestrial birds in the tree, or because they didn't exhibit shifts? I would have expected lots of options, as there are many flightless birds out there.

There are 9 bird species in our dataset that are conventionally regarded as flightless, but to be conservative, we had coded as volant because there is some indication that some can fly even if not routinely and in a limited way (e.g., the Pink-legged rail, Gallirallus insignis, the Giant coot, Fulica gigantea, the Calayan rail, Gallirallus_calayanensis). Re-coding these nine as terrestrial there are now

3 peak-shifts into the Te category (as opposed to 1). The regression analysis for the birds is more or less identical to before although now the Te shifts are significantly > the Vo shifts rather than just numerically greater. We have revised Figure 2 and Supplementary Tables 3 and 4 and changed the text. Please see lines 209-217

Results, Paragraph 12: I'm skeptical of the accuracy of the Reptile aquatic shift frequency result, given that most aquatic reptiles are now extinct (e.g., plesiosaurs, pliosaurs, mosasaurs, countless extinct crocodilians and turtles). Many of these were very large, and I suspect these extinct lineages would have provided evidence for more frequent (and even higher magnitude) shifts. Perhaps this is worth a speculative mention.

We were also puzzled by this result. We've now mentioned the extinct reptiles species, thank you. Please see lines 245-247.

Results, Paragraph 13: I was a little confused by the discussion here about whether terrestrial and aerial changes are constrained (or aquatic ones unconstrained), and specifically the suggestion at the end of the paragraph that constraints likely aren't the reason for the differences in sizes of peak shifts. What is meant here by 'constrained'? My initial interpretation was that you must be talking about selective or functional constraints (in the terminology of Arnold 1992, Am. Nat.), as that would be appropriate for a constraint imposed by an external factor such as buoyancy. However, in explaining why you don't think constraints explain the patterns (in the next two paragraphs), the explanation given is mutational, which is a different type of evolutionary constraint (i.e., this would be Arnold's 'genetic' constraint). Such intrinsic constraints are different from extrinsic constraints such as neutral buoyancy. I think some clarification is needed here. It seems to me that the analyses here are equipped to evaluate the role of mutational constraint, but I'm not sure whether the same can be said for extrinsic constraints.

Agreed. We did mean to be discussing whether the peak shifts are significantly smaller than what could be explained by mutation and drift. We have clarified this. Please see lines 263-265.

Discussion, Paragraph 4-7: I was a little surprised not to see any discussion of the vastly different requirements for supporting body weight against gravity as a reason for the differences in the adaptive landscapes in water, on land, and in air. I agree that different circulatory needs are probably at play, and that aquatic environments probably overwhelming select for fusiform shapes, but supporting the body is probably a key factor here. Water makes it much easier to evolve from small to large, and vice versa, because it buffers the structural support needs of vertebrates (beached whales illustrate this well). The aerodynamic requirements of powered flight work in the other direction, making body size evolution a greater task than in terrestrial relatives, due to the need to ensure that size evolution is accompanied by the concomitant shape evolution necessary to keep the animal airborne.

*We have now added remarks to the Discussion about neutral buoyancy. Please see lines 362-366. Although neutral buoyancy makes it possible to be bigger than on land or in the air and for the reason the reviewer says, we think that the 'easier-to-evolve' in water argument only indirectly explains the larger peak **shifts** of aquatic species in macroevolutionary time (please see above in response to Reviewer 1).*

Discussion, Paragraph 5: Here it is stated that aquatic reptiles have small magnitudes of peak shifts, but Figure 2 suggests this was an error (aquatic reptiles have the largest shift magnitude of all). As noted earlier, I suspect this magnitude is probably an underestimate, given that extant phylogenies poorly represent the full size range of marine reptiles throughout their history.

Thank you. Our mistake, meant to say amphibians, now corrected in the text. Please see lines XXX-XXX.

Methods, Statistical Analyses, Paragraph 2: are the betas indeed actually phylogenetically controlled? Even though they were obtained using a phylogenetic comparative method, might they not still exhibit phylogenetic autocorrelation that should be accounted for, if present?

We analyse the betas as single events that have occurred in a branch. The regression and other statistical analyses expect the betas to be i.i.d. Our proposal mechanism in the Markov chain ensures that every branch has the same probability of attracting a peak shift. A peak shift earlier in a tree does not affect the signal for a peak shift later in the tree because effects are nested. An exception is that a peak shift on one branch of a pair of sister branches normally precludes finding one on the other branch of the pair. But this is a logical problem of inferring ancestral states that affects all comparative methods including parsimony. So it is reasonable to suppose under the null hypothesis that for the β_i *we observe,* $P(\beta_i|\beta_j)=P(\beta_i)$ *for all i,j. The null model also requires that the* β_i *are identically distributed. The prior is constant throughout the tree, this makes their expected variances the same, so under the null they are i.i.d. Other potential influences on the variances (such as habitat or time in the past, etc) are controlled for statistically. The figure below shows the residuals from the vertebrate regression plotted against the predicted values have reasonably homogeneous variance.*

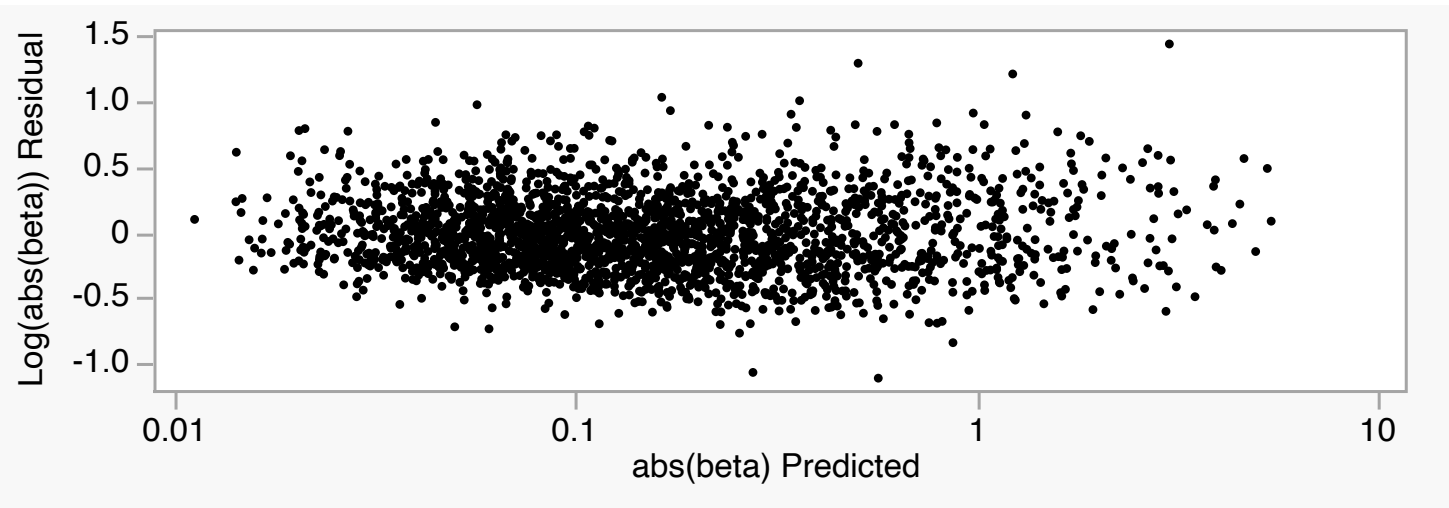

Were the beta effects being accumulated up the tree (or summed at the tips), then the expected variances of these cumulative effects would differ (modelled perhaps as draws from a Poisson with different means), they would have a covariance with other cumulative effects, and both of these would have to be taken into account in a V-C matrix.

In practice, the betas (or their magnitudes, etc) might be associated with regions of the phylogeny as they are here (for example, all volant mammals are bats and bats tend to have smaller betas) but that is an emergent result not part of the model or built-in to the betas. Still, we are aware that it means that everything we say about, for example, flying mammals is confounded with being a bat, and that is why having birds to compare them to is important.

Methods, Stat Analyses, Paragraph 3: "since the present" should be "before the present"

Thank you, now changed. Please see line 520

RESPONSE TO REVIEWERS' COMMENTS

Reviewer #1 (Remarks to the Author):

This is a very satisfying revision of a manuscript I reviewed earlier. I was previously concerned that the paper would be hard to access for people unfamiliar with the fabric model or comparative analyses in general. I feel the authors have elaborated enough on numerous points that the paper will reach a broader audience now. Also, the case for the biological significance of peak-shift size and results about this parameter are clearer. I thank the authors for tolerating my earlier questions and making these changes. I remain interested in how this approach will play out when more complex multivariate data sets are analyzed in a similar way, but I don't think that needs to be dealt with here. This is a very satisfying first step and body size is a compelling univariate trait. I am happy with this ms as it is and congratulate the authors.

Peter Wainwright

Reviewer #1 (Remarks on code availability):

no comments

Thank you to Reviewer 1.

Reviewer #2 (Remarks to the Author):

I was reviewer 2 for the original manuscript.

The authors have prepared a revision, and I think this revised manuscript nicely addresses the issues raised. I have just a few additional comments.

First, in the abstract, it would be helpful to clarify that you infer the population-level variance patterns to arise from evolution on a macroevolutionary adaptive landscape. Otherwise I think the vast majority of readers (including many who will only read the abstract) will assume that your claimed link between micro and macroevolution refers to the traditional concept of intrinsically constrained evolution along genetic lines of least resistance.

We have changed the abstract (lines 33-35) to read: "Within-population trait-variances from independent sources are largest in aquatic, followed by terrestrial then aerial species, consistent with evolution responding to differing macroevolutionary landscapes.

Lines 98-102: I found the text about Brownian motion and optima confusing here. The text describes "a Brownian diffusion process (an unbiased random-walk) augmented by abrupt shifts in the position of optima on the adaptive landscape". Do you also mean the abrupt appearance of an offset optimum? If there optimum were already present, how could evolution be Brownian?

We've revised these lines to read "that characterises the evolution of a phenotypic trait as a function of a Brownian diffusion process (an unbiased random-walk) augmented by the abrupt appearance over time of new optima." (line 103)

343-345 - These lines seem to imply that peak shifts are associated with speciation events (if speciation is decoupled from shifts, the maintenance of genetic identities in lineages occupying

different peaks is much less of a concern). Also, the dominance of allopatry as a speciation mode in vertebrates should largely resolve this concern.

We've replaced that sentence with (lines 343-346)
Weak stabilising selection within populations then means that where successive macroevolutionary peaks yield new species, these peaks must in general be further apart in the trait-space for populations to maintain their genetic identities in the event of interbreeding.

Finally, for the regression of estimated betas:
While I appreciate that beta effects are not expected to be accumulated up the tree, I'm not convinced that this completely rules out the possibility that there could be phylogenetically correlated errors in a regression of them on the proposed independent variables. My remaining concern stems from the fact that these betas are not data, but rather are parameter estimates arising from a fitted model. There will be error associated with their estimation (variances), but because they are jointly estimated, there will also be joint error (covariances) associated with their estimation. Is it not possible that such covariances might reflect the phylogeny? Ultimately I view this as an empirical matter. While I don't have any particularly strong reason to suspect strongly phylogenetically correlated errors for the betas, it seems like something that could be tested, and given that this is a relatively new framework that others will adopt, I think there would be value in looking into this.

We estimated the variance-covariance (VC) matrix of the betas from the behaviour of pairs of betas in the Markov chain. We show below that this matrix can be treated as diagonal, and that the main diagonals are small and approximately homoscedastic. From this it is reasonable to assume that the VC matrix of the errors of regression is also diagonal and homoscedastic. We've added a sentence to the Methods (lines 511-513) explaining why this means it is not necessary to control for the VC matrix of the betas in our analyses and referring the reader to a new section in the Supplementary Information outlining the methodology for investigating the beta VC matrix.

To estimate the beta VC matrix we calculated the correlations among all possible pairs of betas by sampling pairs at widely spaced intervals from our stationary Markov chains. This yields n(n-1)/2 unique correlations (the upper triangle of the symmetric VC matrix). For mammals, for example, there are n=414 betas yielding 85,491 pairwise correlations.

Many of these correlations will be significant by chance. For the mammals, controlling for Type 1 errors at the $\alpha = 0.05$ *level (two-tailed) with the Bonferroni correction yields 300 significant correlations out of the 85,491 pairs (0.35%), the more lenient Hochberg FDR correction, returns 790 (0.92%) significant correlations: over 99% of all correlations are not significant, in keeping with the expectation of a diagonal VC matrix. The other Classes return similar results (SI Table 8). We explain below that these correlations do not affect the final estimates of the betas.*

The Figure shows that the significant correlations (red dots) tend to occur in pairs of betas that are close to each other in the phylogenetic tree whereas non-significant correlations (blue dots) are independent of distance (Figure, left panel; data from all Classses). Were these correlations somehow linked to unaccounted for phylogenetic effects, we would expect the red and blue dots to show a qualitatively similar connection to phylogenetic distance (although red dots will be larger), but they do not.

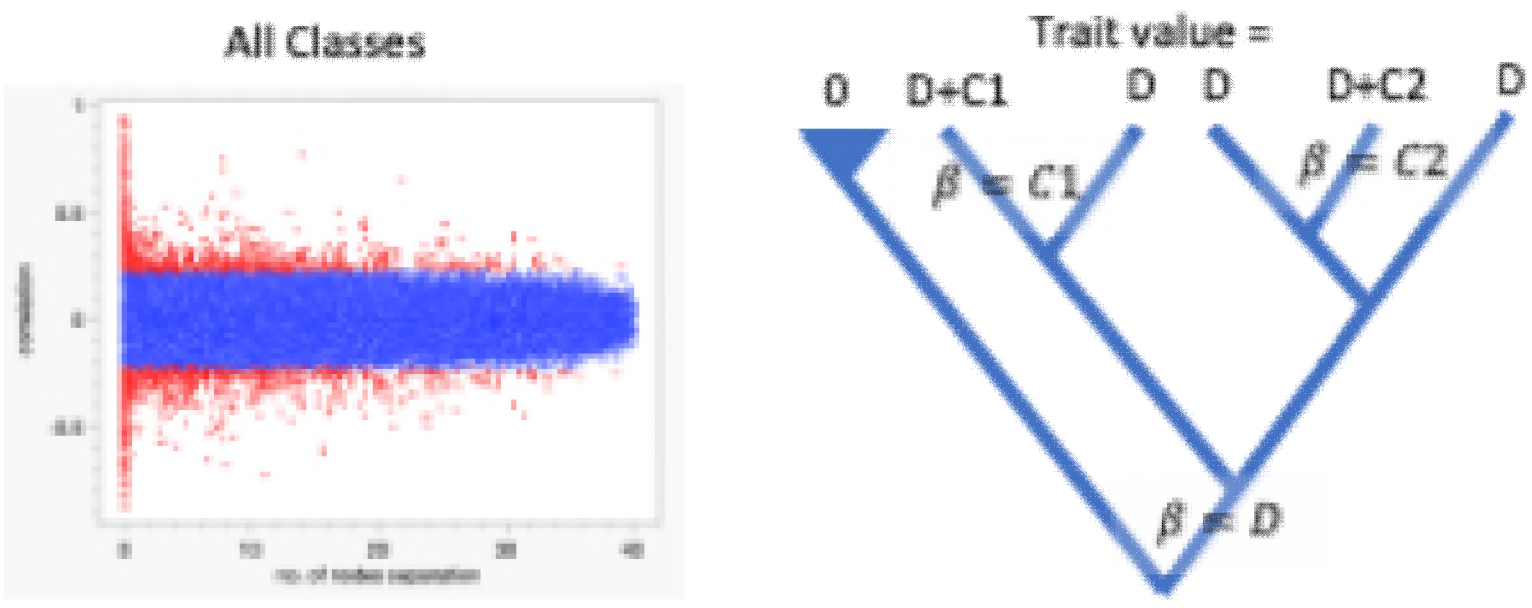

Notes: Left panel: x-axis is a measure of distance between pairs of βs on the phylogenetic tree (the number of nodes separating two βs), y-axis is the correlation between pairs of βs within the Markov chain. Red dots are significant correlations under the more lenient Hochberg criterion, blue are not significant and unrelated to distance (n=651,497 total pairwise correlations; 11,680 = red, 639,817=blue); Right panel: hypothetical tree illustrating how covariation among betas can arise from their estimation in the Markov chain.

The clade of closely related species such as shown in the right panel of the Figure illustrates how the "red" correlations arise from estimation of the joint posterior distributions of pairs of betas. The species within the clade on the right have a trait value of at least D, but two of this clade have trait values of D+C1 and D+C2 (a more distantly related clade of species is depicted as the outgroup triangle, and presumed to take the relative value of zero). This pattern of trait values can be described by proposing an ancestral beta taking the value D, then two further betas taking the values C1 and C2 and placed along the branches leading to these two species. The distance between the ancestral beta and the more derived ones is 1 and 2 respectively.

As the Markov chain runs the proposal mechanism continually updates betas by randomly moving them small amounts (e.g., larger or smaller). Changes to D will tend to be compensated by changes to the two Cs in the opposite direction to maintain their sums = D+C1, D+C2, and creating a negative correlation within the Markov chain. Positive correlations between two C-effect betas will arise because each simultaneously compensates for the same D-effect beta. Both the negative and positive processes are more likely the more closely linked in time two or more events are because they share ancestry and fewer extraneous influences will have intervened. The scenario in the Figure is just one of many possible ways such correlations can arise. Correlations can also extend over greater distance if the $\beta = D$ effect occurs deep in a tree, although they diminish in magnitude with distance.

The correlations arising from estimation within the Markov chain do not affect the betas we analyse. At stationarity the chain estimates the joint posterior distribution of D, C1 and C2. We find the joint mean of the posterior. This averaging removes any correlation that might have arisen within the run. We find average betas this way from six independent Markov chains. As we previously reported in the Methods (line 494-495) the intercorrelation of the average betas taken from pairs of chains is 0.98-0.99. Our final betas are then the average of the six averages. The median coefficient of variation for the betas is 0.019 (IQR = 0.012-0.029), and the variances are only negligibly correlated with their means (r^2 = 0.0003) together indicating small and approximately equal variances along the main diagonal of the VC matrix. From this it is a reasonable assumption that the VC matrix of prediction errors is diagonal and homoscedastic, especially given the high R^2 values of our multiple regressions.
