# Supplementary material for: Convergence in the size and frequency of macroevolutionary peak-shifts on the adaptive landscape in vertebrates
Source: Nat Commun. 2026 Oct 27;17:11208. doi: 10.1038/s41467-026-78289-z (PMC13720082; doi:10.1038/s41467-026-78289-z)
Supplement: Supplementary file 4 [file 41467_2026_78289_MOESM4_ESM.pdf]

Description of Additional Supplementary Files

File Name: Supplementary Data 1

Description: Body size and phylogenetic trees.

File Name: Supplementary Data 2

Description: Ecology and thermoregulation.
